# Supplementary material for: On the incongruence of genotype-phenotype and fitness landscapes
Source: PLoS Comput Biol. 2022 Sep 19;18(9):e1010524. doi: 10.1371/journal.pcbi.1010524 (PMC9521842; doi:10.1371/journal.pcbi.1010524)
Supplement: S1 Appendix — (PDF) [file pcbi.1010524.s001.pdf]

Supplementary material

Derivations

Derivation 1: Derivation of equation 2

The epistasis in the fitness landscape is given by equation 7. We assume σ to be large, such that $(w_i - w_{\text{opt}})^2/\sigma^2 \ll 1\ \forall i \in \{0,1\}^2$, an approximation that holds when selection is weak. Taylor expanding F_i and neglecting higher order terms, we find

$$F_i = \exp\left[-\left(\frac{w_i - w_{\text{opt}}}{\sigma}\right)^2\right] \approx 1 - (w_i - w_{\text{opt}})^2/\sigma^2, \tag{15}$$

where $i \in \{0,1\}^2$. Let $\Delta_i = w_i - w_{\text{opt}}$, then

$$\varepsilon_{\text{f}} \approx \frac{-1}{\sigma^2}\left(\Delta_{00}^2 + \Delta_{11}^2 - \Delta_{01}^2 - \Delta_{10}^2\right) = \frac{-1}{\sigma^2}\left[(\Delta_{00}+\Delta_{11})^2 - 2\Delta_{00}\Delta_{11} - (\Delta_{01}+\Delta_{10})^2 + 2\Delta_{10}\Delta_{01}\right] \tag{16}$$

$$= \frac{-1}{\sigma^2}\left[(\Delta_{00}+\Delta_{11}+\Delta_{01}+\Delta_{10})(\Delta_{00}+\Delta_{11}-\Delta_{01}-\Delta_{10}) - 2(\Delta_{00}\Delta_{11} - \Delta_{10}\Delta_{01})\right]$$

$$= \frac{-1}{\sigma^2}\left[(\Sigma_i\Delta_i)\varepsilon_{\text{gp}} - 2\varepsilon_{m,\Delta}\right]$$

where $\varepsilon_{m,\Delta} = \Delta_{00}\Delta_{11} - \Delta_{10}\Delta_{01} = \varepsilon_{m,\Delta} = (w_{00}-w_{\text{opt}})(w_{11}-w_{\text{opt}}) - (w_{01}-w_{\text{opt}})(w_{10}-w_{\text{opt}}) \implies \varepsilon_{m,\Delta} = \varepsilon_m - w_{\text{opt}}\cdot\varepsilon_{\text{gp}}$, and $\varepsilon_m = w_{00}w_{11} - w_{01}w_{10}$ is the multiplicative epistasis. Substituting for $\varepsilon_{m,\Delta}$, expanding the $\Sigma_i\Delta_i$ term and rearranging gives:

$$\varepsilon_{\text{f}} = \frac{1}{\sigma^2}\left[2\varepsilon_m + (2w_{\text{opt}} - \Sigma_i w_i)\cdot\varepsilon_{\text{gp}})\right] \implies \varepsilon_{\text{f}}\cdot\varepsilon_{\text{gp}} = \frac{1}{\sigma^2}\left[2\varepsilon_m\cdot\varepsilon_{\text{gp}} + (2w_{\text{opt}} - \Sigma_i w_i)\cdot\varepsilon_{\text{gp}}^2\right]. \tag{17}$$

Derivation 2: Derivation of equation 3

The absolute difference between the number of peaks in the genotype-phenotype landscape and the fitness landscape is

$$|p_{\text{f}} - p_{\text{gp}}| = \begin{cases} p_{\text{f}} - p_{\text{gp}}, & \text{if } p_{\text{f}} > p_{\text{gp}} \\ p_{\text{gp}} - p_{\text{f}}, & \text{if } p_{\text{f}} < p_{\text{gp}}. \end{cases} \tag{18}$$

We can rule out the case where $p_{\text{f}} = p_{\text{gp}}$, because the probability of change in the number of peaks tends towards 1 as L increases, so long as w_{opt} is sufficiently far from 1 (Fig 4D). This implies

$$\langle|p_{\text{f}} - p_{\text{gp}}|\rangle = \langle p_{\text{f}} - p_{\text{gp}}\rangle_{p_{\text{f}}>p_{\text{gp}}} P(p_{\text{f}} > p_{\text{gp}}) + \langle p_{\text{gp}} - p_{\text{f}}\rangle_{p_{\text{gp}}>p_{\text{f}}} P(p_{\text{gp}} > p_{\text{f}}), \tag{19}$$

where $\langle p_{\text{f}} - p_{\text{gp}}\rangle_{p_{\text{f}}>p_{\text{gp}}}$ is the conditional mean of $p_{\text{f}} - p_{\text{gp}}$, given that $p_{\text{f}} > p_{\text{gp}}$.

As shown below, the probability of increase in the number of peaks is equal to the probability of decrease in the number of

peaks (Proof 3); i.e., $P(p_{\mathrm{f}} > p_{\mathrm{gp}}) = P(p_{\mathrm{gp}} > p_{\mathrm{f}}) = 0.5$. This implies

$$\langle |p_{\mathrm{f}} - p_{\mathrm{gp}}| \rangle = 0.5(\langle p_{\mathrm{f}} - p_{\mathrm{gp}} \rangle_{p_{\mathrm{f}} > p_{\mathrm{gp}}} + \langle p_{\mathrm{gp}} - p_{\mathrm{f}} \rangle_{p_{\mathrm{gp}} > p_{\mathrm{f}}}). \tag{20}$$

In genotype-phenotype landscapes generated by the House-of-Cards model, the phenotypic values of mutational neighbors are completely uncorrelated, so selection for a low or intermediate phenotypic value generates a fitness landscape in which the fitness values of mutational neighbors are also completely uncorrelated. Thus, the distribution of the number of peaks is the same for the genotype-phenotype landscape and the fitness landscape. Moreover, p_{f} is independent of p_{gp} because p_{f} depends upon the number of genotypes with phenotypes close to the optimal phenotypic value, and this number is independent of the number of genotypes with phenotypes close to 1. Therefore, p_{gp} and p_{f} are independent and identically distributed (i.i.d) random variables. Now as $L \to \infty$, the distribution of the number of peaks in the House-of-Cards model tends to a normal distribution [1] with mean $\frac{a^L}{(a-1)\cdot L+1}$ and variance $\tau^2 = \frac{a^L\cdot((a-1)\cdot L-(a-1))}{2((a-1)\cdot L+1)^2}$ [2], where a is the number of alleles at each site and here $a = 2$. This implies the distribution of the random variable $p_{\mathrm{f}} - p_{\mathrm{gp}}$ is also normally distributed with mean zero and variance $2\tau^2$; i.e.,

$$p_{\mathrm{f}} - p_{\mathrm{gp}} := x \sim \mathcal{N}(0, 2\tau^2), \tag{21}$$

which is symmetric about zero, implying $\langle p_{\mathrm{f}} - p_{\mathrm{gp}} \rangle_{p_{\mathrm{f}} > p_{\mathrm{gp}}} = \langle p_{\mathrm{gp}} - p_{\mathrm{f}} \rangle_{p_{\mathrm{gp}} > p_{\mathrm{f}}}$. Thus,

$$\langle |p_{\mathrm{f}} - p_{\mathrm{gp}}| \rangle = \langle p_{\mathrm{f}} - p_{\mathrm{gp}} \rangle_{p_{\mathrm{f}} > p_{\mathrm{gp}}} = \frac{1}{\sqrt{2\pi}\tau'} \int_0^\infty x \cdot \exp\left[-\frac{x^2}{2\tau'^2}\right] dx = \frac{\tau}{\sqrt{\pi}} = \frac{1}{\sqrt{\pi}} \sqrt{\frac{a^L((a-1)\cdot L-(a-1))}{2((a-1)\cdot L+1)^2}}, \tag{22}$$

where $\tau'^2 = 2\tau^2$.

So, for $a = 2$, as in our biallelic landscapes,

$$\lim_{L\to\infty} \langle |p_{\mathrm{f}} - p_{\mathrm{gp}}| \rangle = \sqrt{\frac{2^L \cdot (L-1)}{2\pi(L+1)^2}}. \tag{23}$$

Proofs

Proof 1: Selection for w_{opt} cannot transform a simple sign epistasis motif into a reciprocal sign epistasis motif

In order to have a reciprocal sign epistasis motif:

- No two adjacent arrows can point in the same direction.
- No two parallel arrows can point in the same direction.

Because sign epistasis motifs cannot be cyclic, the two conditions above imply one another. The simple sign epistasis motif determines the complete rank ordering of the phenotypic values, unlike the other two motifs, which only give a partial ordering. Therefore, upon selecting for any w_{opt}, at least two arrows in the genotype-phenotype landscape remain the same as in the fitness landscape or reverse in direction together. This results in a pattern of arrows in the fitness landscape that always contradicts both the above mentioned conditions for reciprocal sign epistasis (see S1 Fig). ■

Proof 2: The phenotype-fitness map changes a reciprocal sign epistasis motif into a no sign epistasis motif or a simple sign epistasis motif with equal probability

The reciprocal sign epistasis motif does not determine the complete rank ordering of the four phenotypic values. In particular, it does not determine the ordering of the two peaks and the two valleys. Let the phenotypic value of the higher of the two peaks be w_{p1} and that of the lower peak be w_{p2}. Similarly, let the labels w_{v1} and w_{v2} refer to the higher and lower valleys, respectively. By design, these values lie on the real number line between 0 and 1. We define the "neighbourhood" of each of the phenotypic values as the region on the number line around the phenotypic value that is closer to it than to any other phenotypic value (S2 Fig). For transformation of the reciprocal sign epistasis motif into one of the other two motifs, w_{opt} must lie in the neighbourhood of either w_{p2} or w_{v1} and it must be second closest to the other (i.e., $F_{\text{p2}} > F_{\text{v1}} > \text{rest}$ or $F_{\text{v1}} > F_{\text{p2}} > \text{rest}$, where F_i represents the fitness corresponding to the phenotype w_i). If not, the reciprocal sign epistasis motif will be retained. So, for any w_{opt}, we only consider those configurations that satisfy this condition. Amongst these configurations, both phenotypic values are equally likely to be the closest to w_{opt}. Let's call the probability of either event happening $P_0 = P(F_{\text{v1}} > F_{\text{p2}} > \text{rest}) = P(F_{\text{p2}} > F_{\text{v1}} > \text{rest})$. We denote the transition probabilities amongst motifs as $P(x|rs)$, where x represents either the no sign epistasis motif, ns, or the simple sign epistasis motif, ss, and rs represents the reciprocal sign epistasis motif. Now, for the no sign epistasis motif to emerge, the fitness values of the peaks or valleys need to be "separated", while for the simple sign epistasis motif to emerge, they need to be "interspersed" (see S2), giving us the following:

$$P(ns|rs) = P(F_{\text{v1}} > F_{\text{p2}} > \text{rest}) \cdot P(F_{\text{p1}} > F_{\text{v2}}) + P(F_{\text{p2}} > F_{\text{v1}} > \text{rest}) \cdot P(F_{\text{p1}} < F_{\text{v2}}), \tag{24}$$

and

$$P(ss|rs) = P(F_{\text{v1}} > F_{\text{p2}} > \text{rest}) \cdot P(F_{\text{p1}} < F_{\text{v2}}) + P(F_{\text{p2}} > F_{\text{v1}} > \text{rest}) \cdot P(F_{\text{p1}} > F_{\text{v2}}). \tag{25}$$

Finally, using $P_0 = P(F_{\text{v1}} > F_{\text{p2}} > \text{rest}) = P(F_{\text{p2}} > F_{\text{v1}} > \text{rest})$ gives:

$$P(ns|rs) = P_0 \cdot P(F_{\text{p1}} > F_{\text{v2}}) + P_0 \cdot P(F_{\text{p1}} < F_{\text{v2}}) \text{ and } P(ss|rs) = P_0 \cdot P(F_{\text{p1}} < F_{\text{v2}}) + P_0 \cdot P(F_{\text{p1}} > F_{\text{v2}}) \implies P(ns|rs) = P(ss|rs). \blacksquare \tag{26}$$

Proof 3: For House-of-Cards genotype-phenotype landscapes, selection for low or intermediate phenotypic values is equally likely to increase or decrease the number of peaks in the fitness landscape, relative to the genotype-phenotype landscape

For any number of loci L, the probability that selection for a low or intermediate phenotypic value causes an increase in the number of peaks in the fitness landscape, relative to the genotype-phenotype landscape, is

$$P_{\text{inc}} = \Sigma_{i>j} P(p_{\text{f}} = i | p_{\text{gp}} = j) \cdot P(p_{\text{gp}} = j), \tag{27}$$

whereas the probability of a decrease in the number of peaks is

$$P_{\text{dec}} = \Sigma_{i>j} P(p_{\text{f}} = j | p_{\text{gp}} = i) \cdot P(p_{\text{gp}} = i), \tag{28}$$

where $i, j \in \{1, 2, ...2^{L-1}\}$, $P(p_{\text{f}} = j | p_{\text{gp}} = i)$ is the probability of having j peaks in the fitness landscape after selection for w_{opt} given the genotype-phenotype landscape has i peaks, and $P(p_{\text{gp}} = i)$ is the probability of i peaks in the genotype-phenotype landscape. The number of peaks in the fitness landscape, p_{f}, is independent of the number of peaks in the genotype-phenotype landscape, p_{gp}, because p_{f} depends upon the number of genotypes with phenotypes close to the optimal phenotypic value, and this number is independent of the number of genotypes with phenotypes close to 1. This implies:

$$P(p_{\text{f}} = i | p_{\text{gp}} = j) \cdot P(p_{\text{gp}} = j) \; = \; P(p_{\text{f}} = i) \cdot P(p_{\text{gp}} = j), \tag{29}$$

and

$$P(p_{\text{f}} = j | p_{\text{gp}} = i) \cdot P(p_{\text{gp}} = i) \; = \; P(p_{\text{f}} = j) \cdot P(p_{\text{gp}} = i). \tag{30}$$

Moreover, p_{gp} and p_{f} are identically distributed; i.e., $P(p_{\text{gp}} = i) = P(p_{\text{f}} = i)$

$$\implies P_{\text{inc}} = P_{\text{dec}} \blacksquare \tag{31}$$

Notes

Note 1: Number of maxima in the plot of $\langle|p_{\mathbf{f}} - p_{\mathbf{gp}}|\rangle$ versus $w_{\mathbf{opt}}$ for additive biallelic landscapes

Any biallelic, additive landscape can be relabelled such that the genotype with the lowest phenotypic value is represented by 000.....0 and the genotype with the highest phenotypic value by 111.....1. Moreover, the phenotypic values can be re-scaled such that genotype 000.....0 has phenotypic value zero and genotype 111.....1 has phenotypic value one. This yields a monotonically increasing genotype-phenotype landscape, wherein the phenotypic effects of every single mutant is larger than zero and they add up to 1; i.e.,

$$w_{100...0} + w_{010...0} + ... + w_{000...1} = 1 \tag{32}$$

where $w_{100...0}$ is the phenotypic value of a single mutant with the mutation at the 1st site, and so on. Equation 32 implies that the vector of single mutant phenotypes belongs to a standard $L-1$-simplex[‡].

Two-locus case

A two-locus biallelic fitness landscape can have a maximum of two peaks. Because the additive genotype-phenotype landscape has only one peak, selection for a low or intermediate phenotypic value can increase the number of peaks in the fitness landscape, relative to the genotype-phenotype landscape, by at most one. Let the fitness of one of the single mutants be x, forcing the fitness of the other single-mutant to be $1-x$. Due to the relabelling of the genotype-phenotype landscape, a two-peaked fitness landscape is only possible when the two single-mutants are peaks. This will occur when the two single-mutants have phenotypes that are closer to w_{opt} than the phenotypes of both genotypes 00 and 11. This leads to the following conditions on x:

$$1 - 2 \cdot w_{\text{opt}} < x < 2 \cdot w_{\text{opt}}, \forall w_{\text{opt}} < 0.5 \tag{33}$$

and

$$2 \cdot w_{\text{opt}} - 1 < x < 2 - 2 \cdot w_{\text{opt}}, \forall w_{\text{opt}} > 0.5. \tag{34}$$

From Eqs 33 and 34, we can infer the shape of the $L = 2$ curves in Fig 4A and 4B. Specifically, for $w_{\text{opt}} < 0.25$ and $w_{\text{opt}} > 0.75$, we get the absurd condition $0.5 < x < 0.5$, which implies there is no possible value of x that will lead to two peaks. Further, for $w_{\text{opt}} = 0.5$, $0 < x < 1$, which implies there will always be two peaks. For $0.25 \leq w_{\text{opt}} < 0.5$ or $0.5 < w_{\text{opt}} \leq 0.75$, the fitness landscape can have either one or two peaks.

Three-locus case

[‡] An n dimensional simplex is the convex hull of $n+1$ affinely independent points. E.g., a 0-simplex is a point, a 1-simplex is a line, a 2-simplex is a triangle, and a 3-simplex is a tetrahedron.

Here, the single-mutant phenotypic values belong to the 2-simplex formed by the points (0,0,1), (0,1,0) and (1,0,0). Let (w_1, w_2, w_3) be an arbitrary point in the simplex, where w_i is the phenotypic value of the single mutant with a mutation at the ith site and $i \in \{1,2,3\}$. Although, a three-locus, biallelic landscape can have at most four peaks, the transformed additive genotype-phenotype landscapes can have at most three, due to the correlations between the phenotypic values. So for instance, if the three single mutants become peaks, then the triple mutant cannot be a peak because the double mutants will be assigned a higher fitness than the triple mutant. This is caused by correlations in the additive landscape, which makes the double-mutants closer in phenotypic value to the single-mutants than the triple mutant. Thus, it is easy to see that the only three peak configuration possible is one in which either all the single mutants become peaks or all the double mutants become peaks. For the former to be the case, each single mutant phenotypic value w_i must satisfy the following conditions:

$$(w_i - w_{\text{opt}})^2 < (0 - w_{\text{opt}})^2 \tag{35}$$

and

$$(w_i - w_{\text{opt}})^2 < (1 - w_j - w_{\text{opt}})^2 \ \forall j \neq i. \tag{36}$$

While Eq. 35 necessitates the single mutant fitness values to be higher than the wild type fitness, Eq. 36 requires them to be higher than the neighbouring double mutant fitness values. On average, $w_i = 1/3 \ \forall i$, so on average, the simplex area enclosed by the above conditions will be maximised when $w_{\text{opt}} = 1/3$. A similar analysis can be done for the case when the three double mutants become peaks and in this case, the area is maximised when $w_{\text{opt}} = 2/3$. This explains the presence of the two maxima in Fig 4A at $w_{\text{opt}} = 1/3$ and $w_{\text{opt}} = 2/3$. Further, the dip at $w_{\text{opt}} = 0.5$ can be explained by the following argument: Assuming the single mutant phenotypic values are of the type $(x, y, 1-x-y)$, the double mutant phenotypic value will be of the type $(x+y, 1-x, 1-y)$. At $w_{\text{opt}} = 0.5$, the wild type and triple mutant immediately become minima and leave an opportunity for the intermediate mutants to become peaks. However, if one of the single mutants (say with fitness x) is closest in fitness to 0.5, the other two cannot be peaks because the double mutant formed by combining these two mutations will have the same fitness i.e., $|x - 0.5| = |(1-x) - 0.5|$. So at $w_{\text{opt}} = 0.5$, there are two peaks after selection for a low phenotypic value and therefore the net change is 1, which is smaller than what we see at $w_{\text{opt}} = 1/3, 2/3$.

Four and more locus cases

We could explicitly derive the shape of the curves for the $L = 2$ and $L = 3$ cases because it was easy to visualise the simplices. For $L \geq 4$, we can form an intuition about the expected result, by considering the average additive fitness landscape, which guides the behavior of the average change in the number of peaks. Because the only randomness in these landscapes arises from the effect of single mutations, which as described above, belong to the L-dimensional simplex, all we need to look at is the average L-dimensional vector of single mutation effects $\langle \vec{w}_1^L \rangle$ and the deviations around it. These random vectors belong to an $L-$dimensional Dirichlet distribution with all the concentration parameters equal to one. It can be shown by symmetry

that $\langle \vec{w}_1^L \rangle = (1/L, 1/L, , , 1/L)$. Therefore, on average, the fitness of a mutant with n mutations is n/L. As w_{opt} increases from zero, the number of peaks will gradually start to increase as w_{opt} gets closer the value of the fitness of single mutants. Then as w_{opt} approaches $1/L$, we encounter the first maxima because at this w_{opt}, all single mutants get to be peaks. Next, there is a slight decline due to competition between the single and double mutants as peaks (because these are mutational neighbours and therefore cannot both be peaks), followed by the emergence of the second maxima at $2/L$ when all the double mutants become peaks, and so on. Naturally, because the number of genotypes with n mutations increases as n reaches the value $L/2$, the height of the maxima increases accordingly. Thereafter, we observe a fall in $\langle |p_{\text{f}} - p_{\text{gp}}| \rangle$. Therefore, for a genotype of length L, there should be $L-1$ maxima in the plot of $\langle |p_{\text{f}} - p_{\text{gp}}| \rangle$ vs. w_{opt}. However, the maxima are closely spaced and therefore merge into one another and cannot be distinguished from each other.

Note 2: Evolution on NK landscapes

S3A and S3C Fig shows the average length of a greedy adaptive walk $\langle l \rangle$ in NK landscapes with $L=5$ and $L=8$. For all $K < L-1$, $\langle l \rangle$ is minimised when $w_{\text{opt}} = 0.5$, whereas for $K = L-1$ (House-of-Cards landscapes), $\langle l \rangle$ is independent of w_{opt}. This can be understood by first considering additive genotype-phenotype landscapes ($K=0$), wherein genotypes with a phenotype $w \approx 0.5$ are by definition, approximately located half-way between the wild-type and its antipodal sequence. Selecting for $w_{\text{opt}} = 0.5$ thus minimizes the average length of a greedy adaptive walk to these peak genotypes. As K increases, correlations among the phenotypes of mutationally-neighboring genotypes are gradually broken and the landscape becomes more rugged. Yet, genotypes with a phenotype $w \approx 0.5$ tend to remain near the center of the landscape. When K is increased to $L-1$, the phenotypes of mutationally-neighboring genotypes become completely uncorrelated and monotonic increases in phenotype along mutational paths to peak genotypes become exceedingly rare, rendering $\langle l \rangle$ independent of w_{opt}. Thus the "U"-shape of the curve of $\langle l \rangle$ versus w_{opt} flattens as K increases from 0 to $L-1$. Interestingly, so long as $K > 0$, the average height of the peak reached by a greedy adaptive walk is also maximized at $w_{\text{opt}} = 0.5$, and these local peaks are >97% the height of the global peak for $\sigma = 1$ (S7 Fig), an observation we explain below.

We next simulated deterministic mutation-selection dynamics. S3B and S3D Fig show the change in mean fitness at equilibrium when selecting for w_{opt}, relative to fitness at equilibrium when selecting for $w_{\text{opt}} = 1$, for $\sigma = 0.5$ and $\mu = 0.1$ (see S8 Fig for other values of σ and μ). This change is always positive, increases with L and K, and tends to be maximized at $w_{\text{opt}} = 0.5$ when $K > 0$. The latter occurs because the optimal phenotype $w_{\text{opt}} = 0.5$ is, on average, closer to all other phenotypic values in the landscape. For instance for $K = L-1$, the sum $\Sigma_{i=1}^{2^L} (w_i - w_{\text{opt}})^2$ is minimized when $w_{\text{opt}} = \Sigma_{i=1}^{2^L} w_i / 2^L = \langle w \rangle = 0.5$. Thus, selecting for $w_{\text{opt}} = 0.5$ creates a fitness landscape with higher average fitness than fitness landscapes under selection for any other w_{opt}. This also explains why the average height of local adaptive peaks reached is maximized at $w_{\text{opt}} = 0.5$ (S7 Fig). In sum, these results show that selection for an intermediate phenotypic value decreases the length of a greedy adaptive walk and increases mean fitness at equilibrium, despite increasing the overall ruggedness of the fitness landscape, relative to the genotype-phenotype landscape.

References

1. Baldi P & Rinott Y. Asymptotic normality of some graph-related statistics. *J. Appl. Probab.* **26**, 171–175 (1989).

2. Macken CA & Perelson AS. Protein evolution on rugged landscapes. *Proc. Natl. Acad. Sci.* **86**, 6191–6195, (1989).
