# Supplementary figures and images for: On the incongruence of genotype-phenotype and fitness landscapes
Source: PLoS Comput Biol. 2022 Sep 19;18(9):e1010524. doi: 10.1371/journal.pcbi.1010524 (PMC9521842; doi:10.1371/journal.pcbi.1010524)

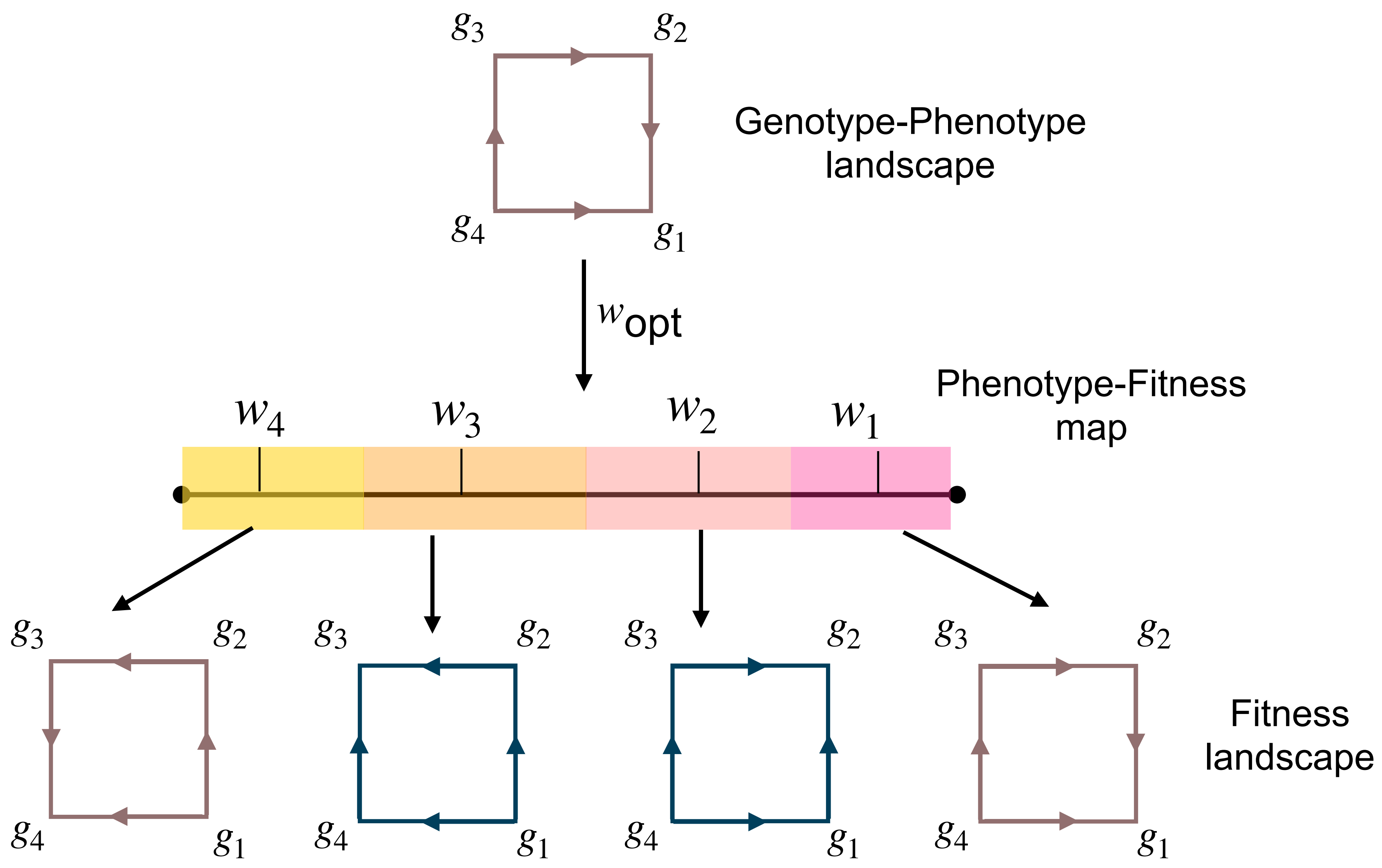

Supplement: S1 Fig — A simple sign epistasis motif (shown in brown) cannot be changed into a reciprocal sign epistasis motif, for any wopt. The no sign epistasis motif is shown in blue. The remaining colours represent the neighbourhood of the phenotypic values (wi) corresponding to each genotype (gi), where i ∈ {1, 2, 3, 4} and the genotypes are labelled in descending order, such that the genotype with the highest phenotypic value is labelled 1, and so on. The four bottom arrows point to the transformed motifs when wopt belongs to the neighbourhood from which the arrow emanates. (PDF) [file pcbi.1010524.s002.pdf]

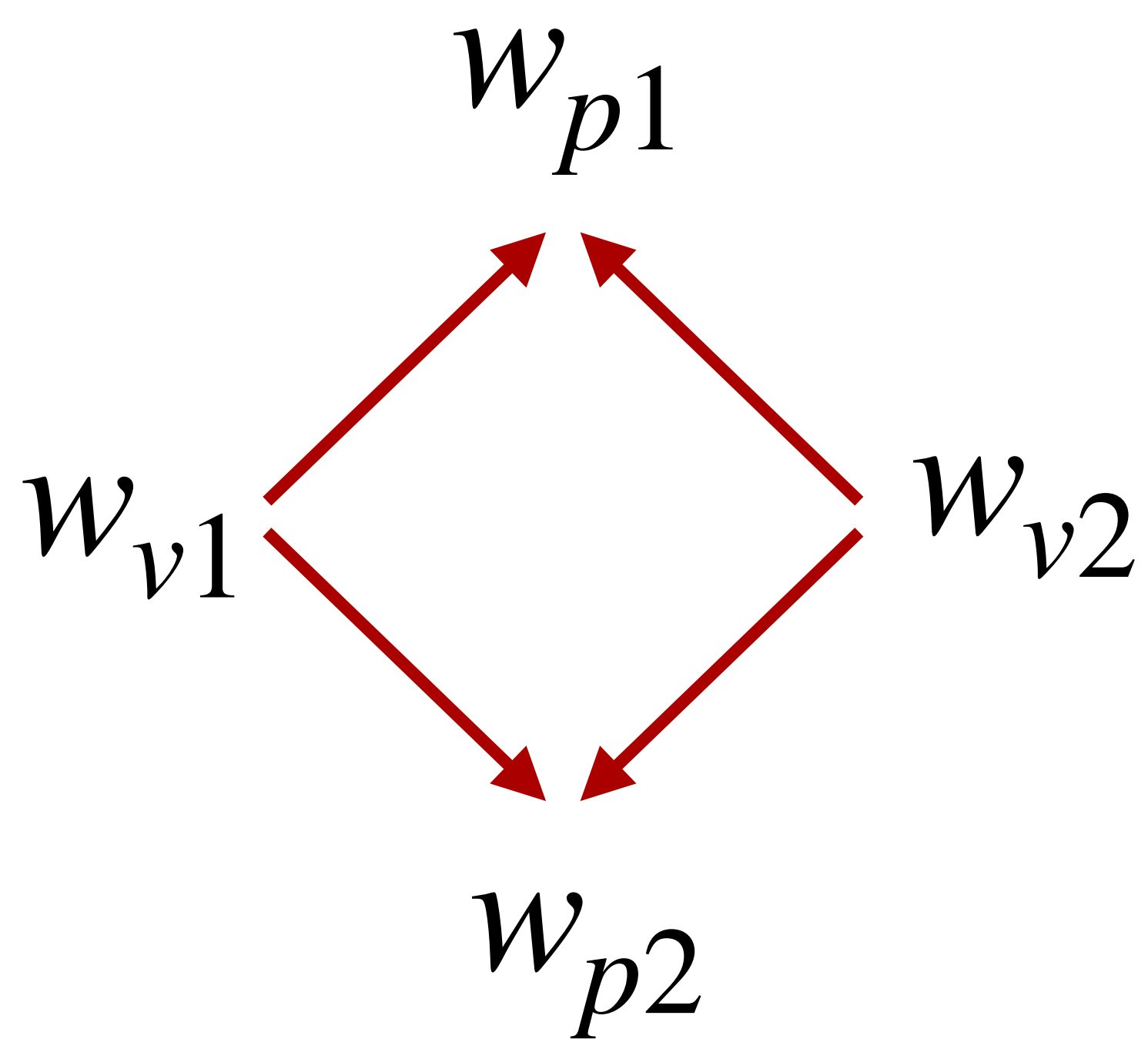

Reciprocal sign epistasis

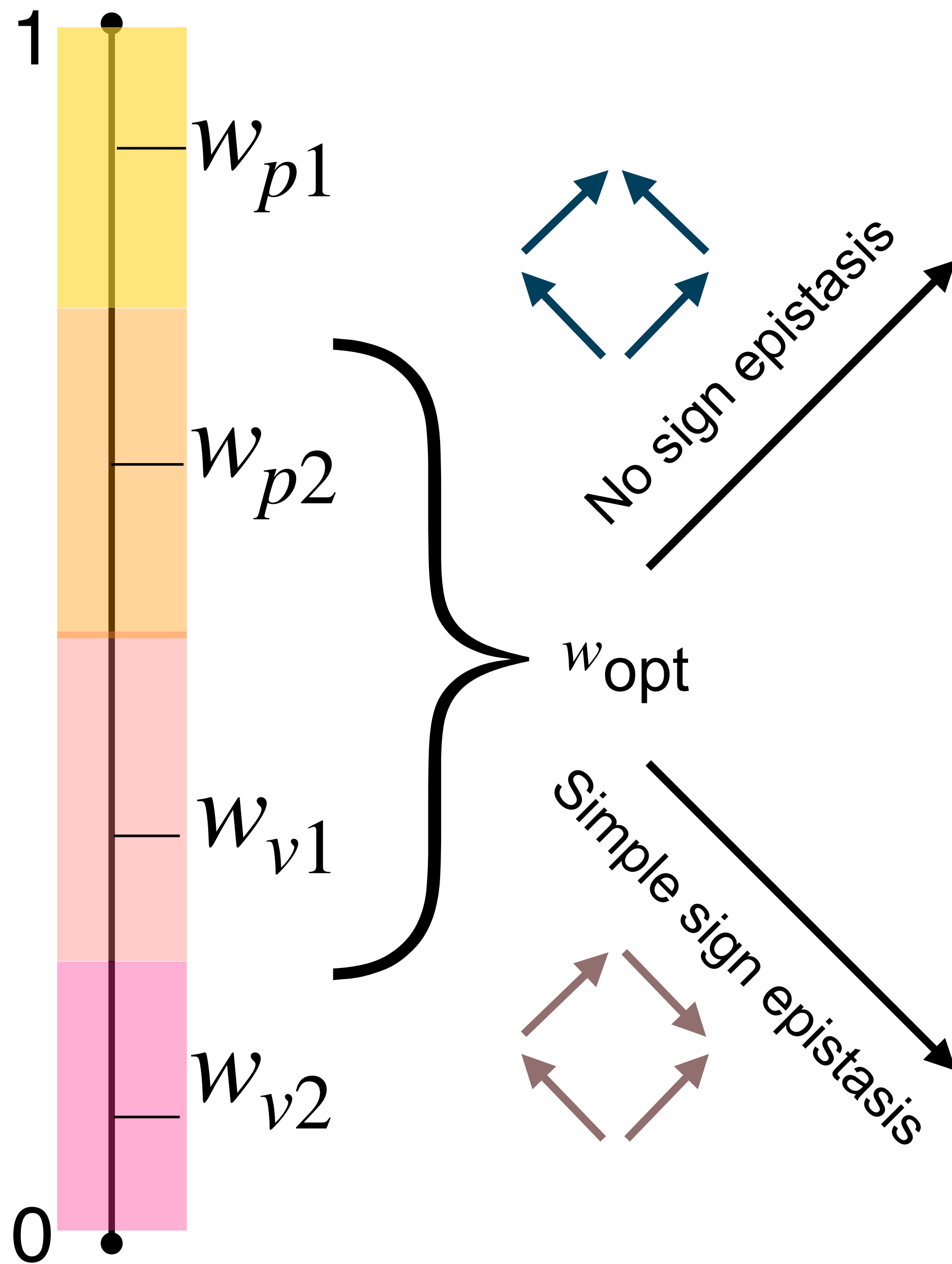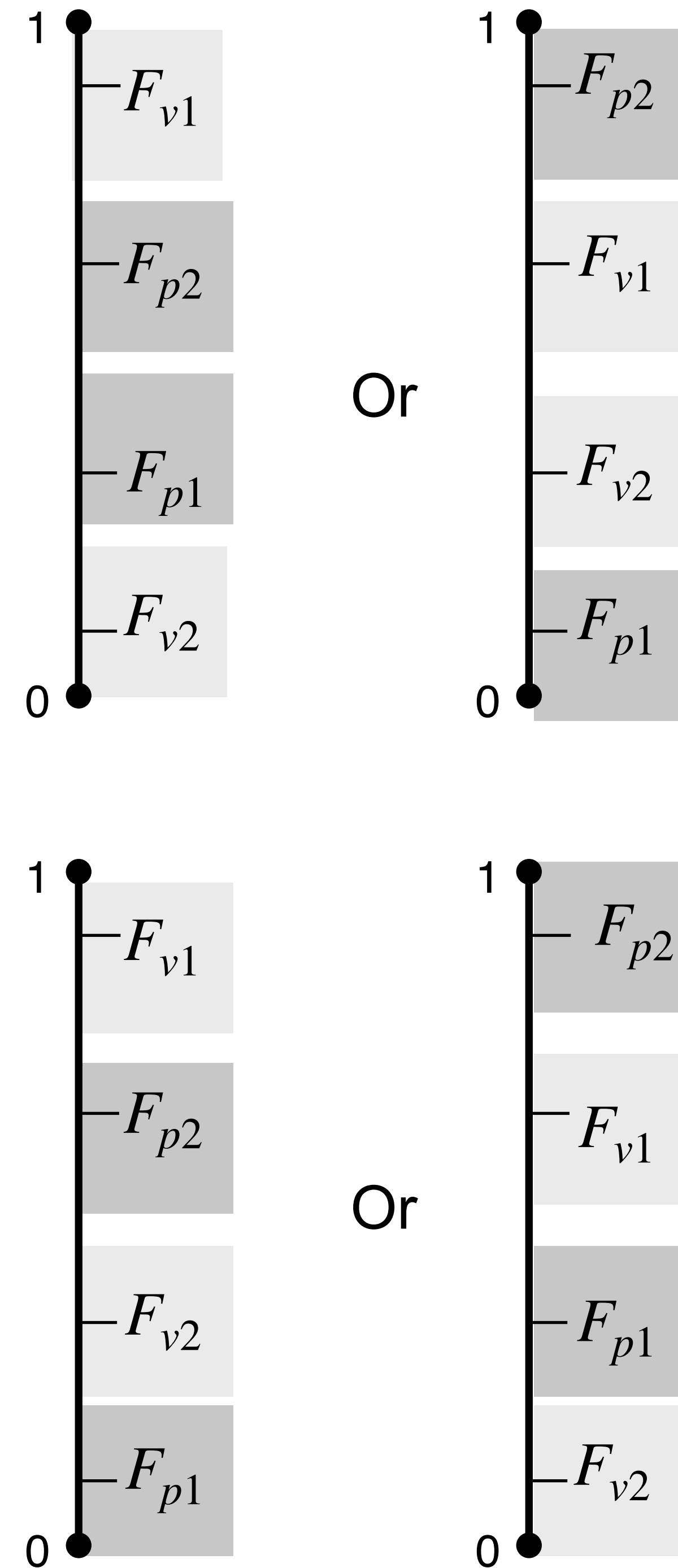

Supplement: S2 Fig — Selection for wopt transforms the reciprocal sign epistasis motif (shown in red) into the no sign epistasis motif (shown in blue) and the simple sign epistasis motif (shown in brown) with equal probability. The neighbourhood of each phenotypic value is shown in a different colour. For the no sign epistasis motif to emerge (top), the fitness values need to be “separated”, while for the simple sign epistasis motif to emerge (bottom), the fitness values need to be “interspersed”. (PDF) [file pcbi.1010524.s003.pdf]

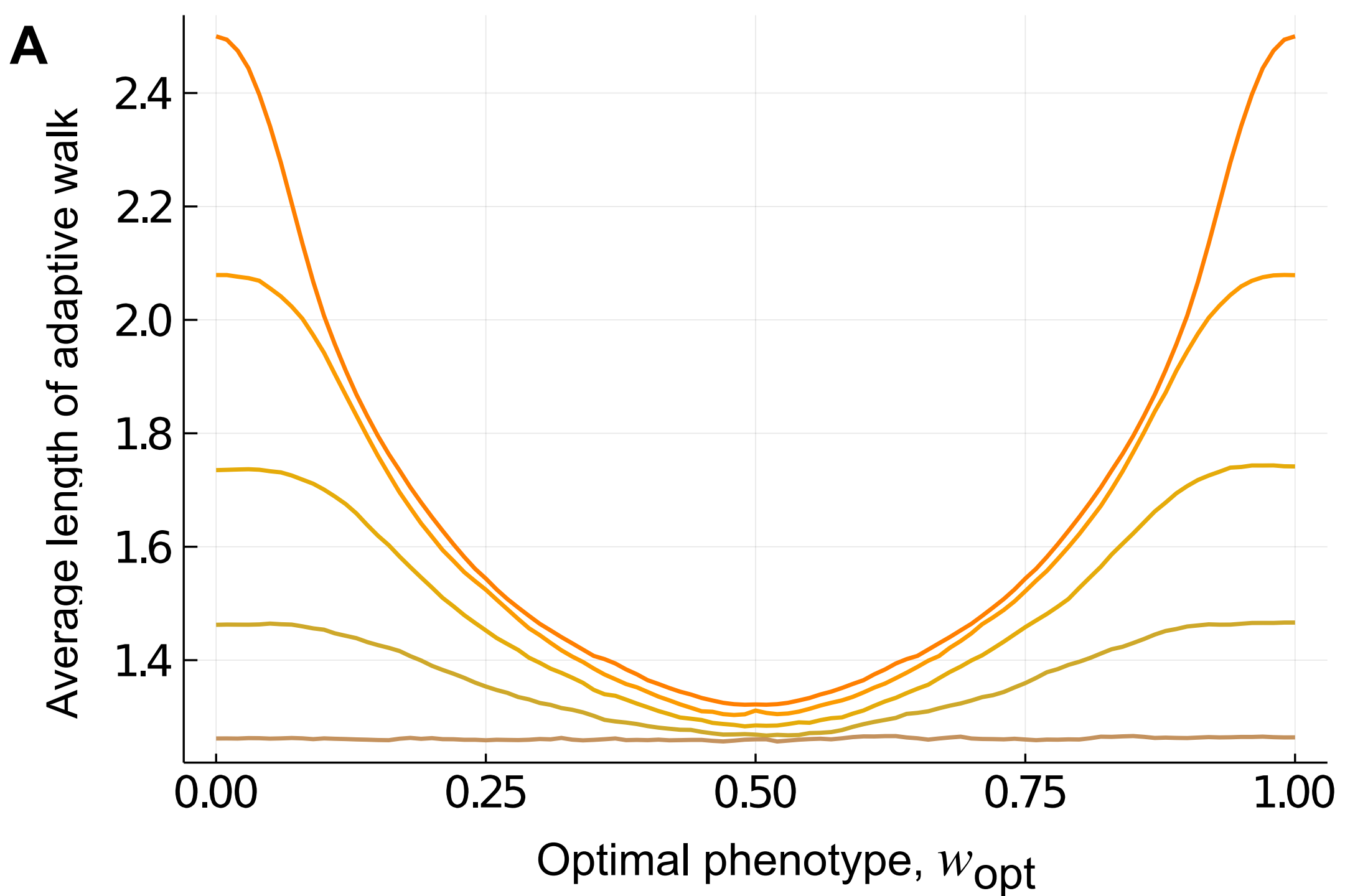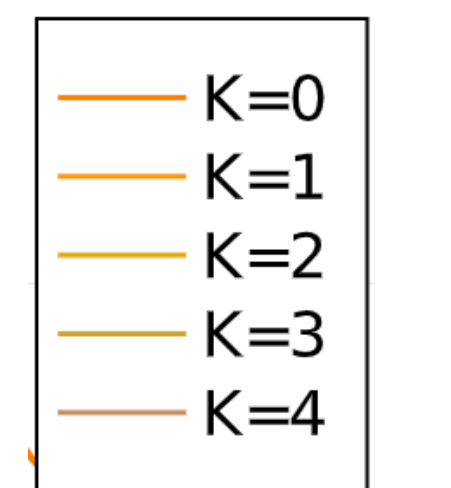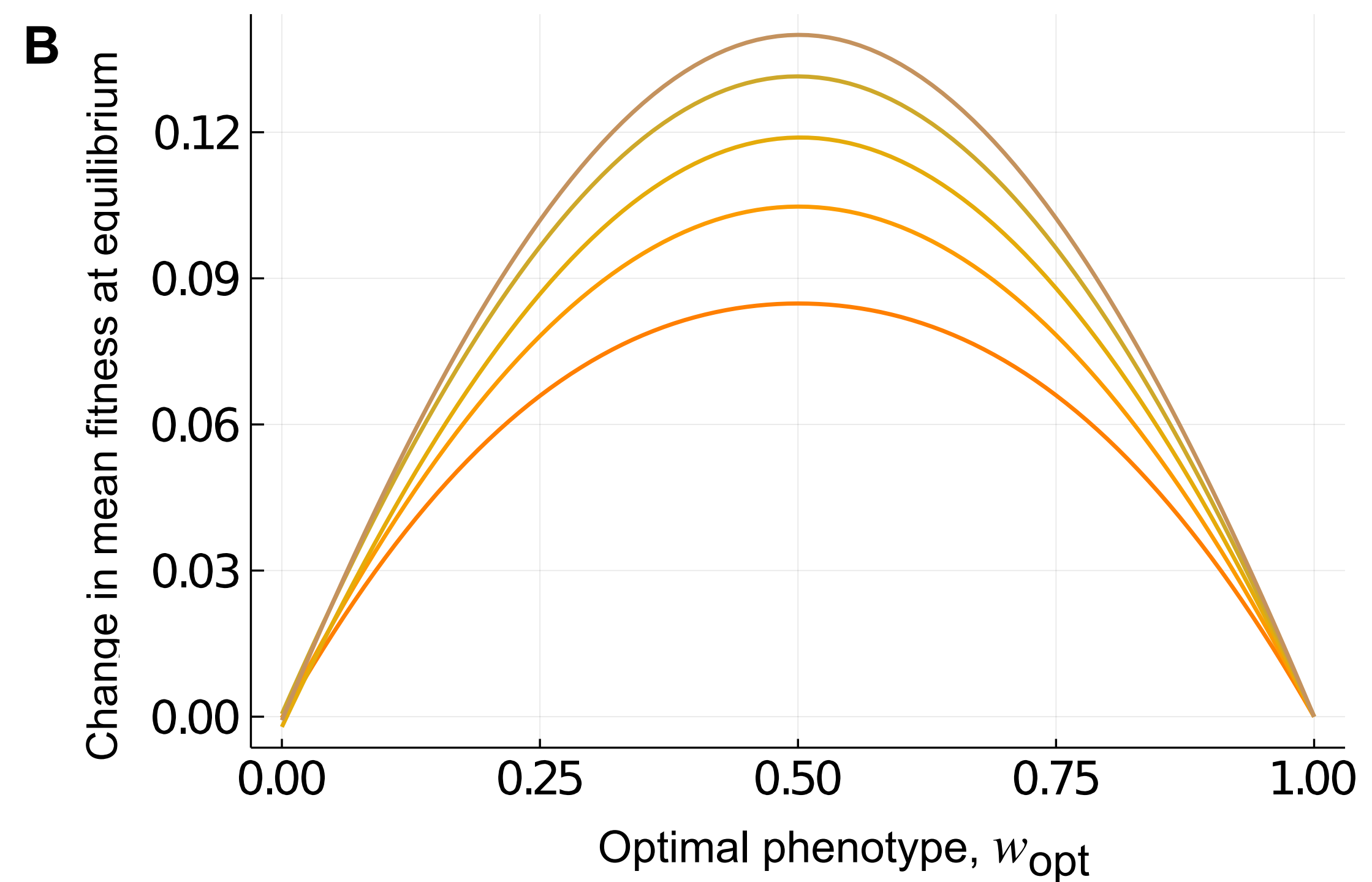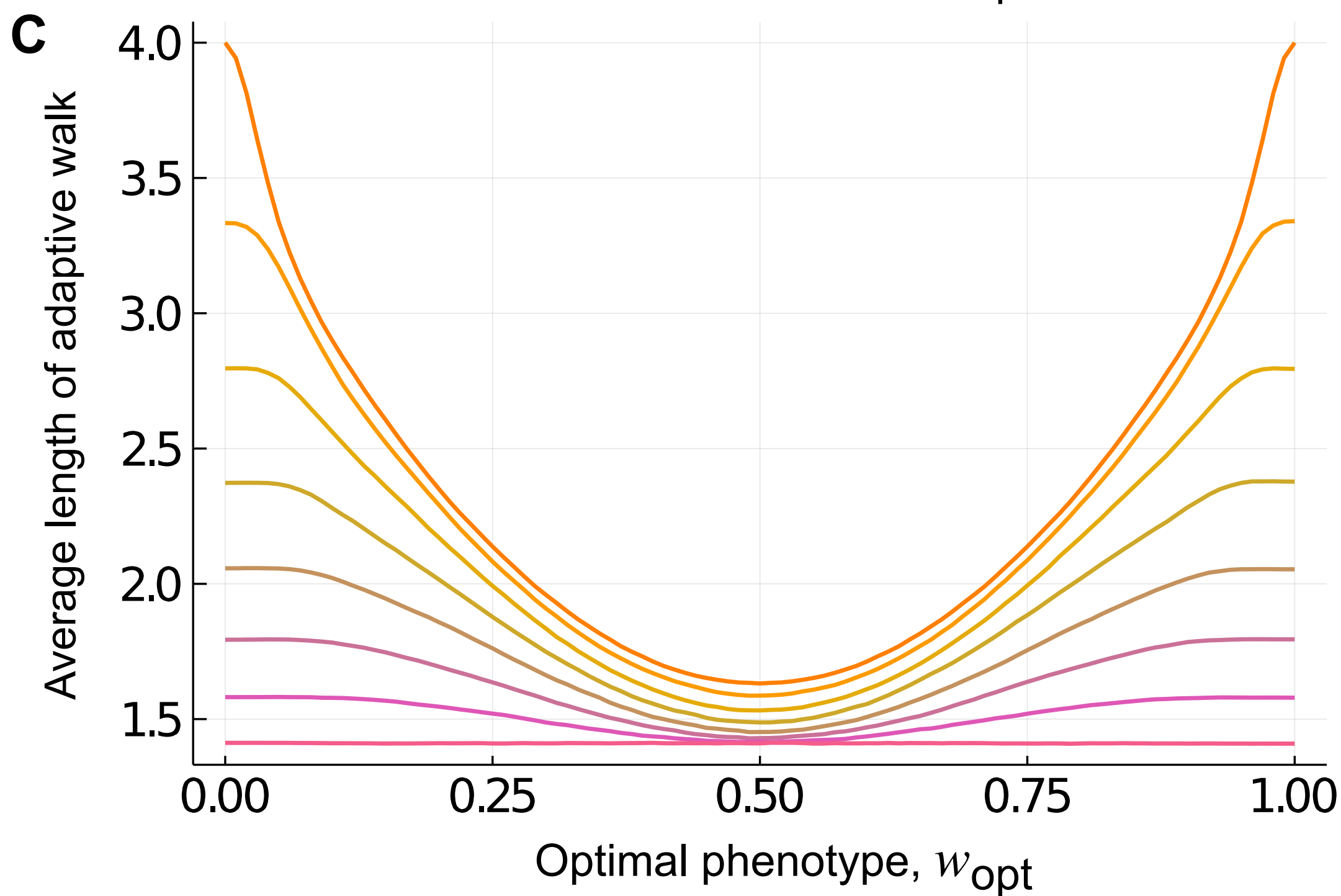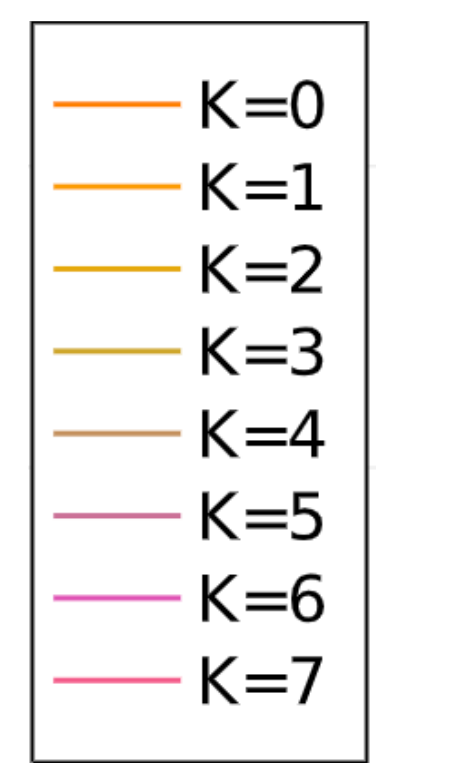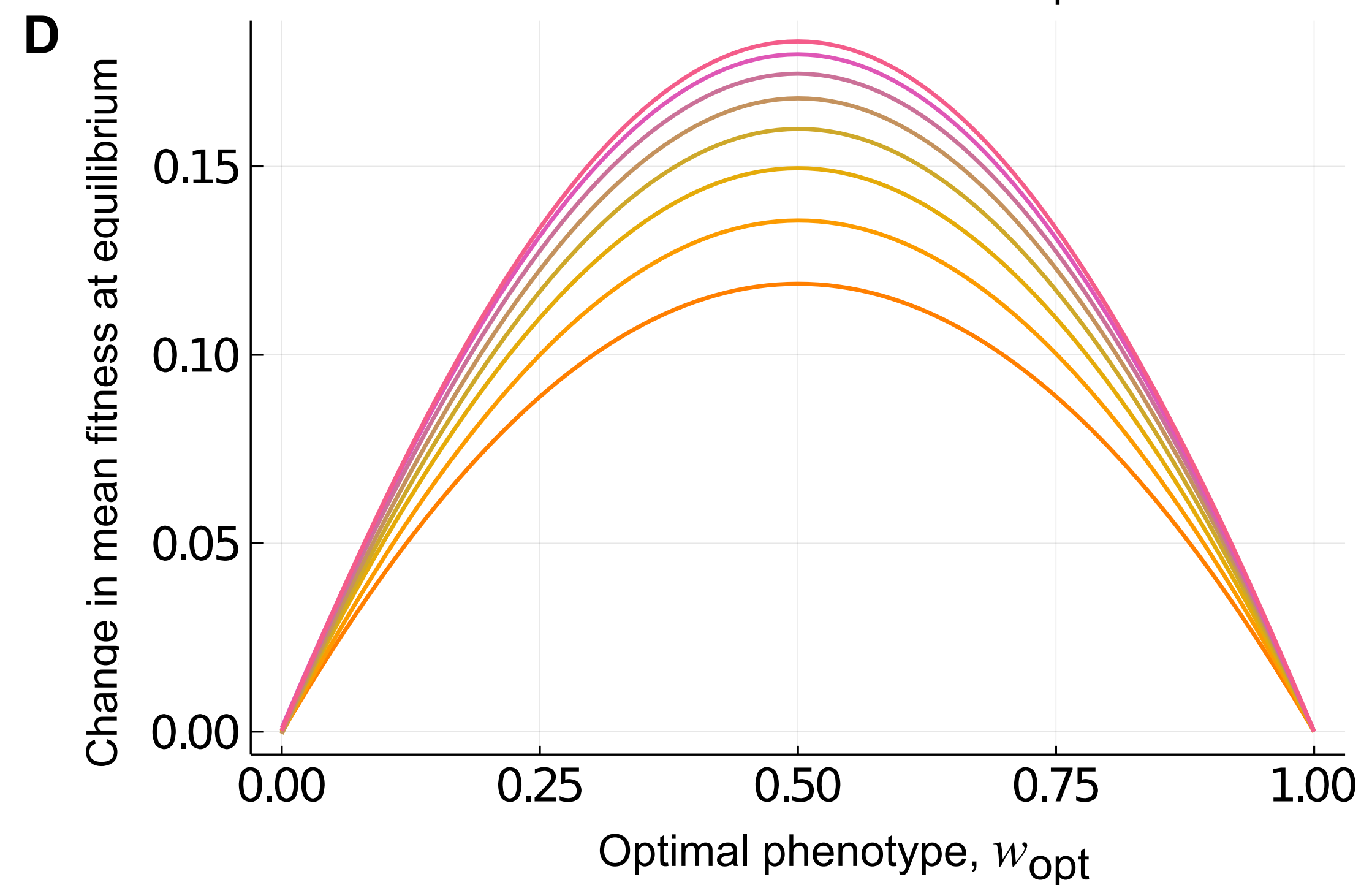

Supplement: S3 Fig — (A,C) The average length of a greedy adaptive walk and (B,D) the change in mean fitness at equilibrium, relative to fitness at equilibrium when selecting for wopt = 1, are shown for (A,B) L = 5 and (C,D) L = 8. In (B,D), μ = 0.1 and σ = 0.5. (PDF) [file pcbi.1010524.s004.pdf]

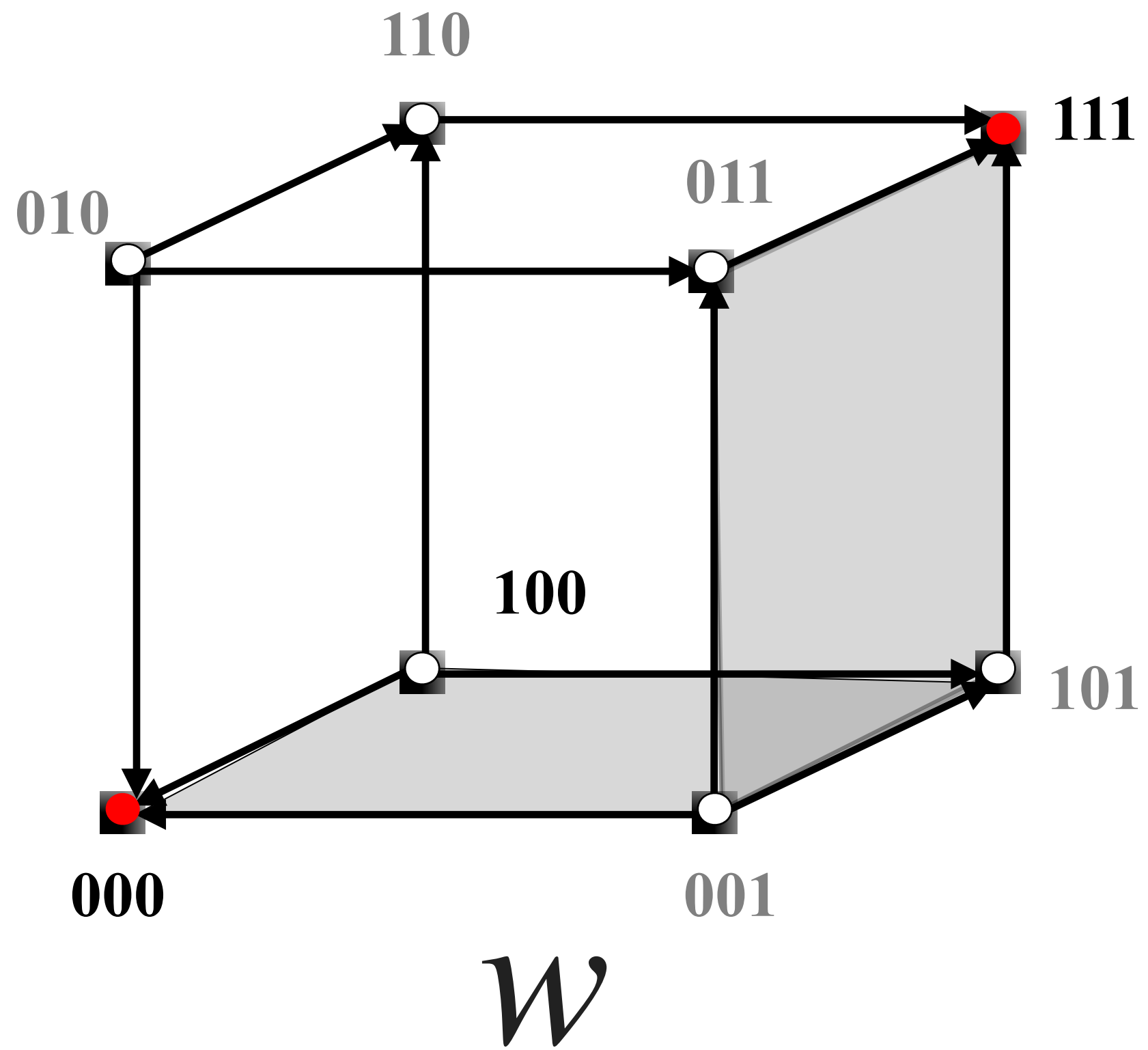

$$w_{opt} = 0$$

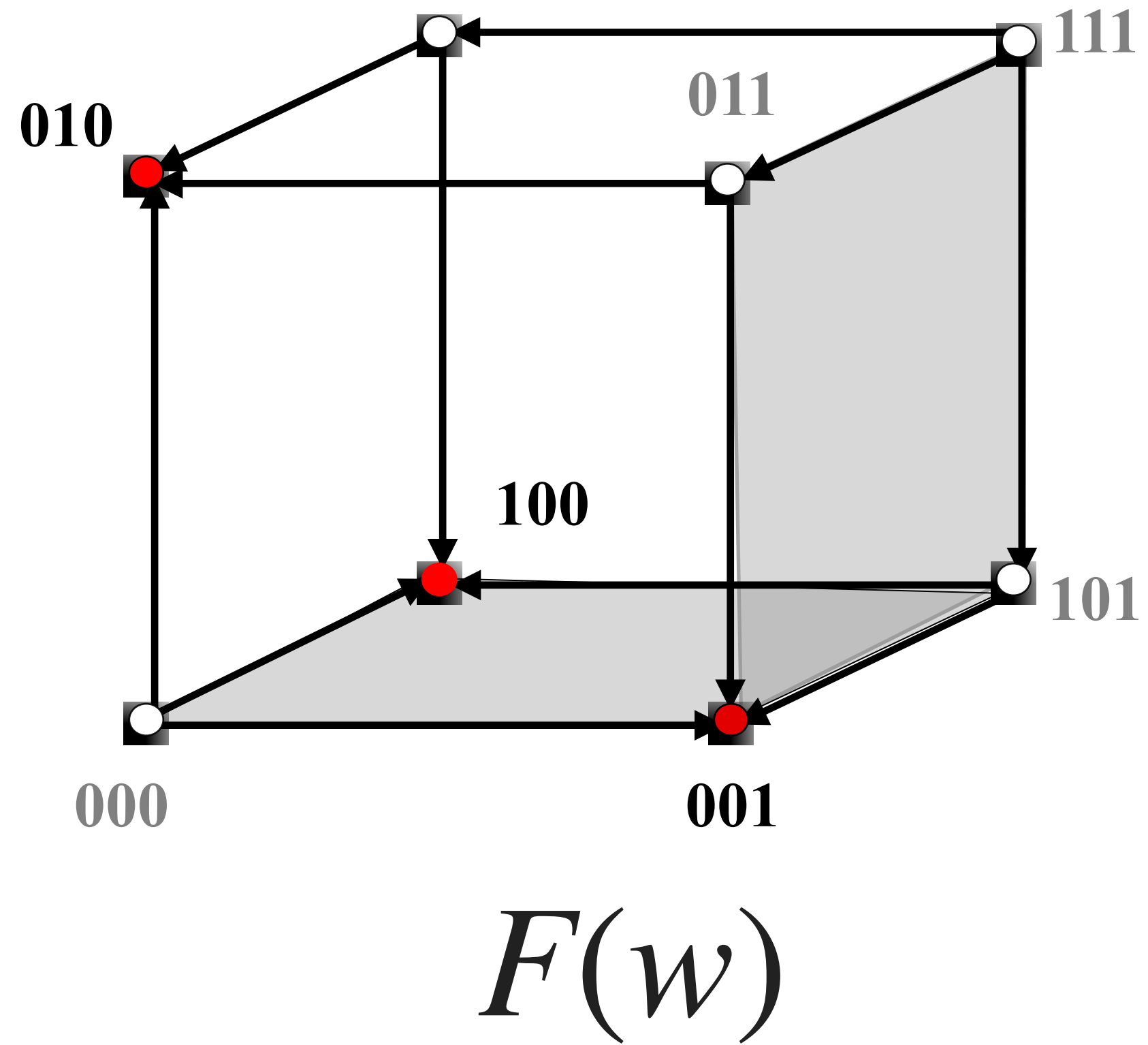

Supplement: S4 Fig — Selection for wopt = 0 does not change the type of epistasis motif for any “square” in the fitness landscape, relative to the genotype-phenotype landscape, yet it can change the number and location of peaks. To understand how, consider that any two adjacent faces of the hypercube (e.g., gray faces above) are sufficient to determine whether the genotypes on their common edge are peaks or not. After selecting for wopt = 0, the type of epistasis motif does not change in the adjacent faces, yet the number and location of the peaks on their common edge does change. Peak genotypes are shown in red. Arrows point from lower to higher phenotypic or fitness values. (PDF) [file pcbi.1010524.s005.pdf]

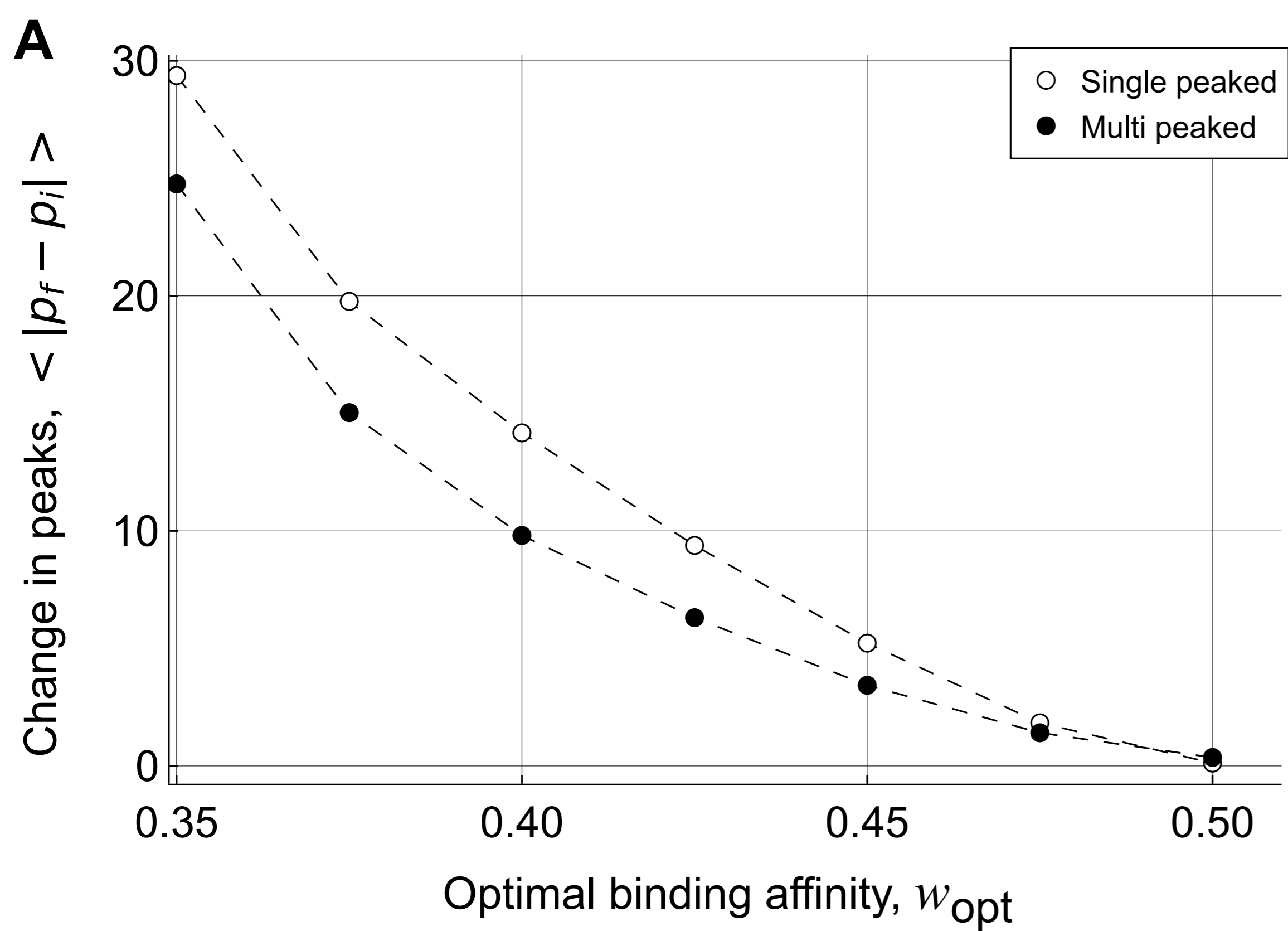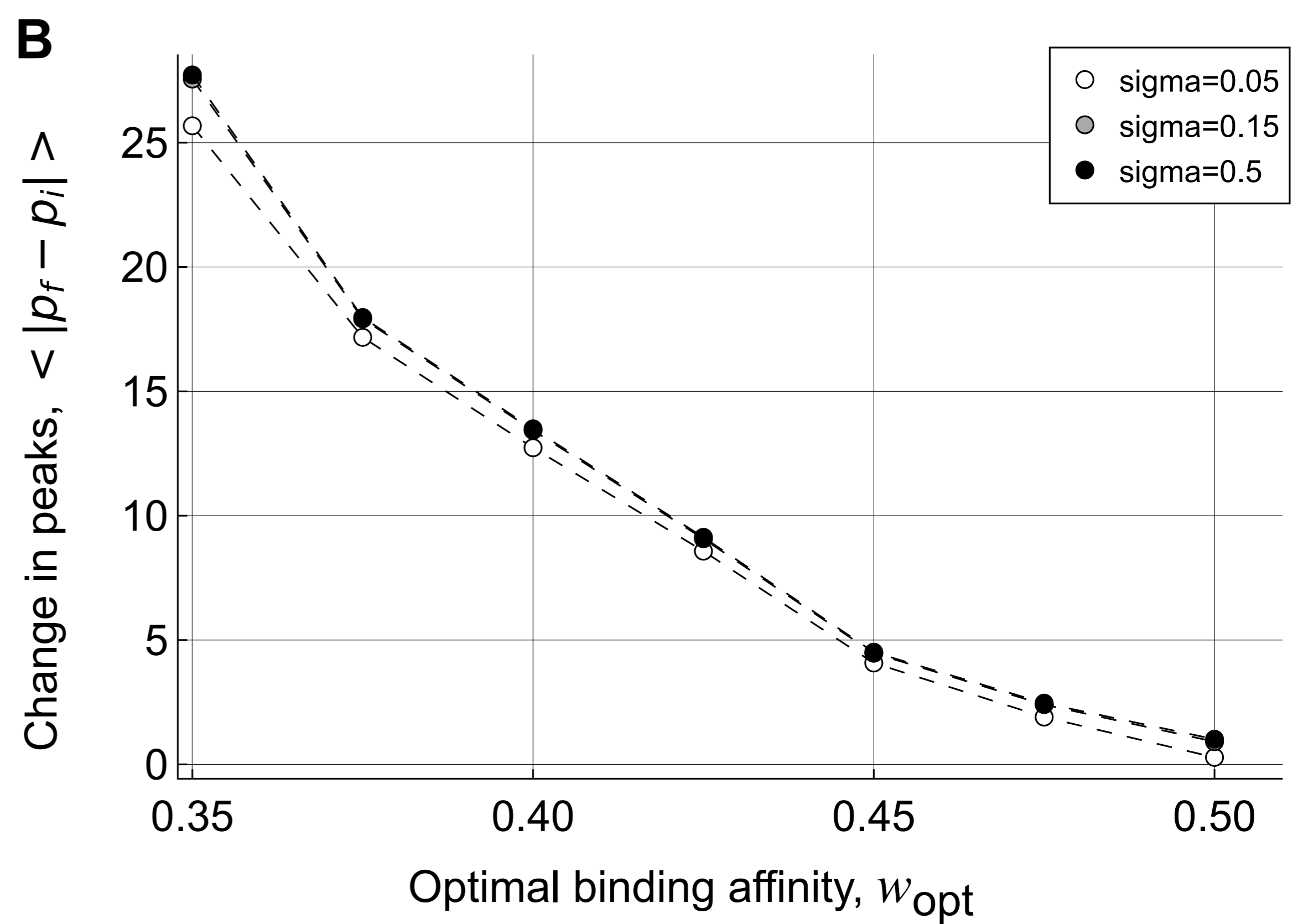

Supplement: S5 Fig — Mean absolute change in the number of peaks in the fitness landscape relative to the genotype-phenotype landscape, shown in relation to wopt, for (A) single-peaked vs. multi-peaked genotype-phenotype landscapes and (B) three values of σ. (PDF) [file pcbi.1010524.s006.pdf]

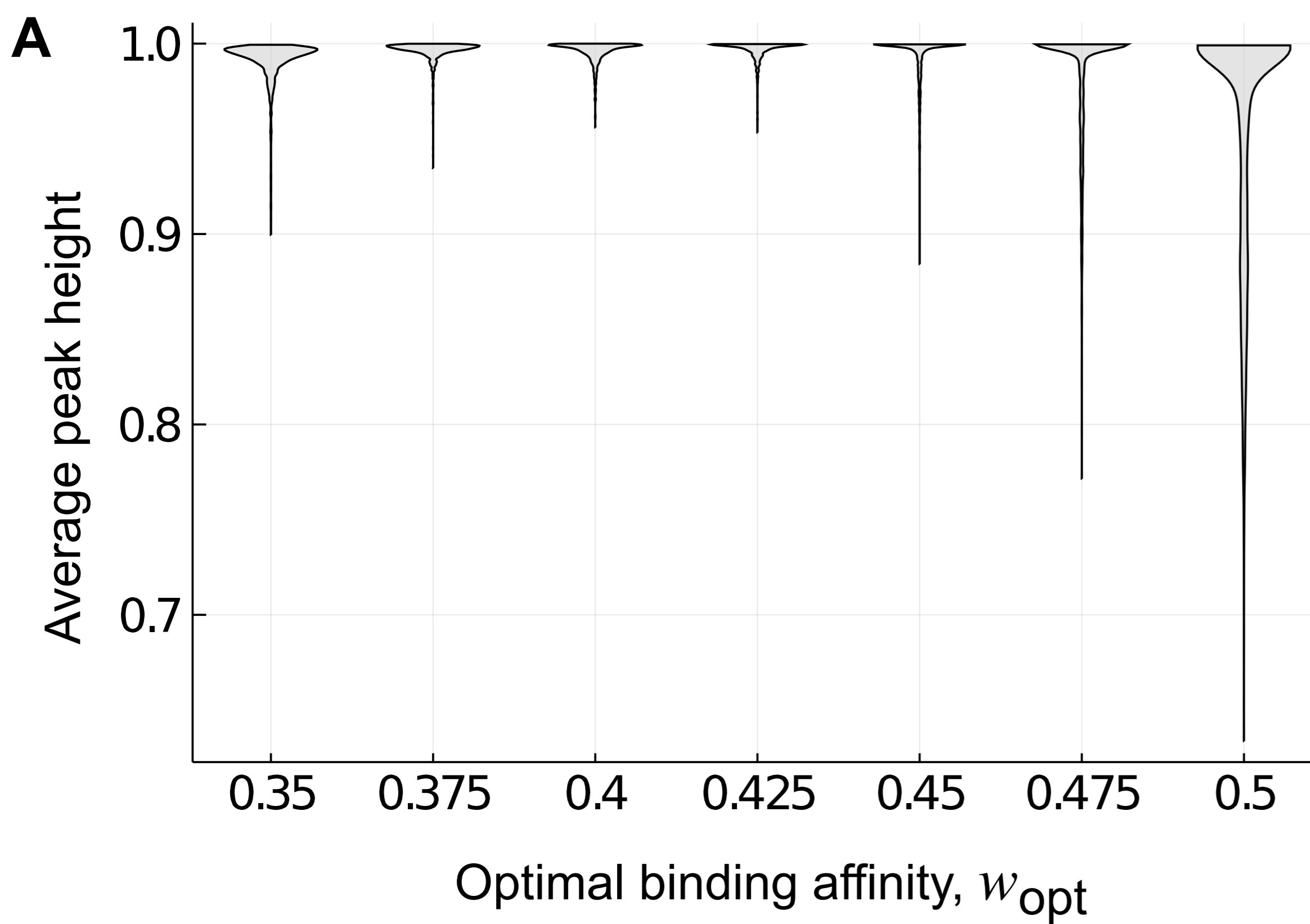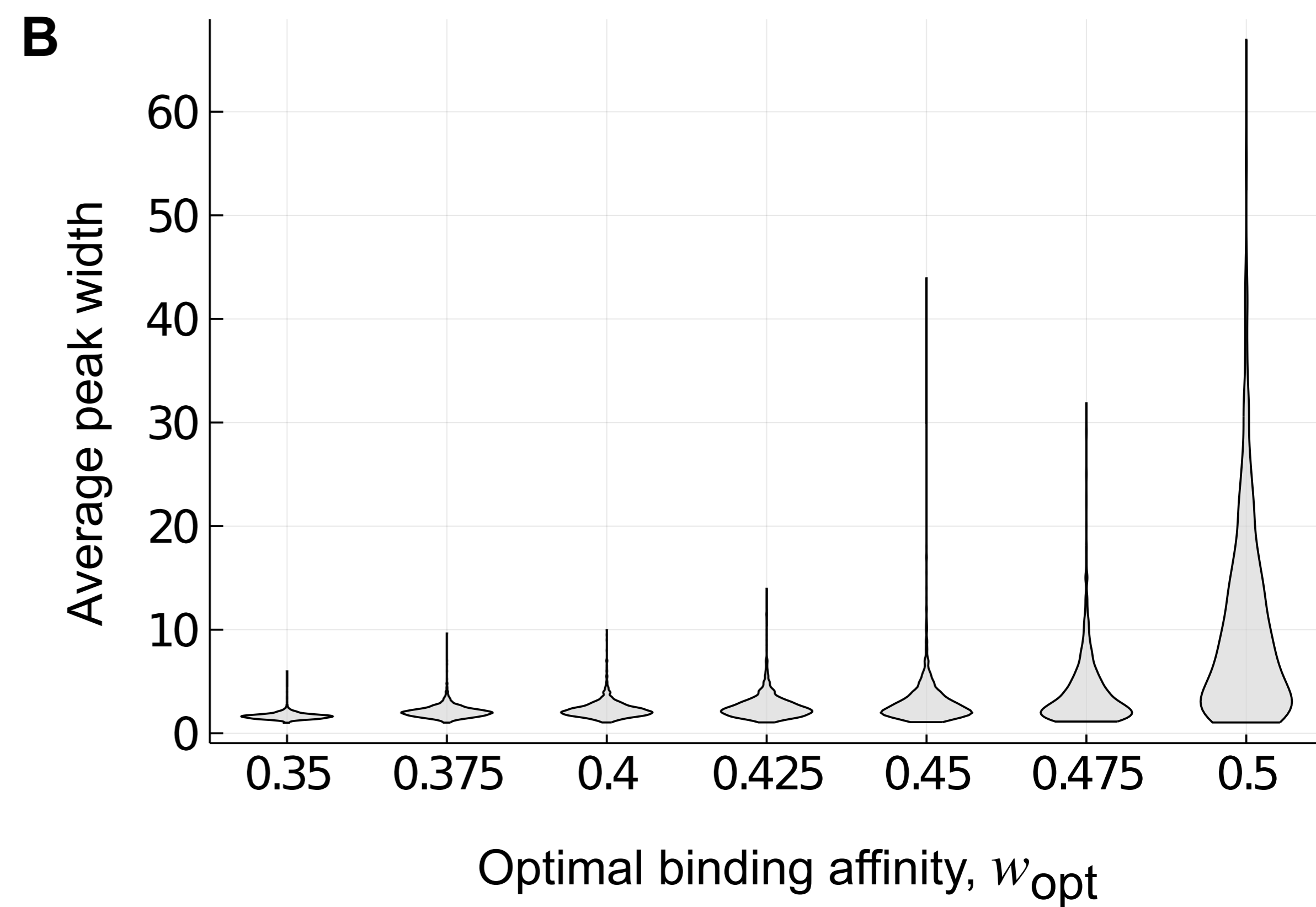

Supplement: S6 Fig — Average peak (A) height and (B) width for 1,137 empirical landscapes, shown in relation to the optimal binding affinity wopt. Violin plots show the distribution across the landscapes for each wopt. Data include both local and global peaks. (PDF) [file pcbi.1010524.s007.pdf]

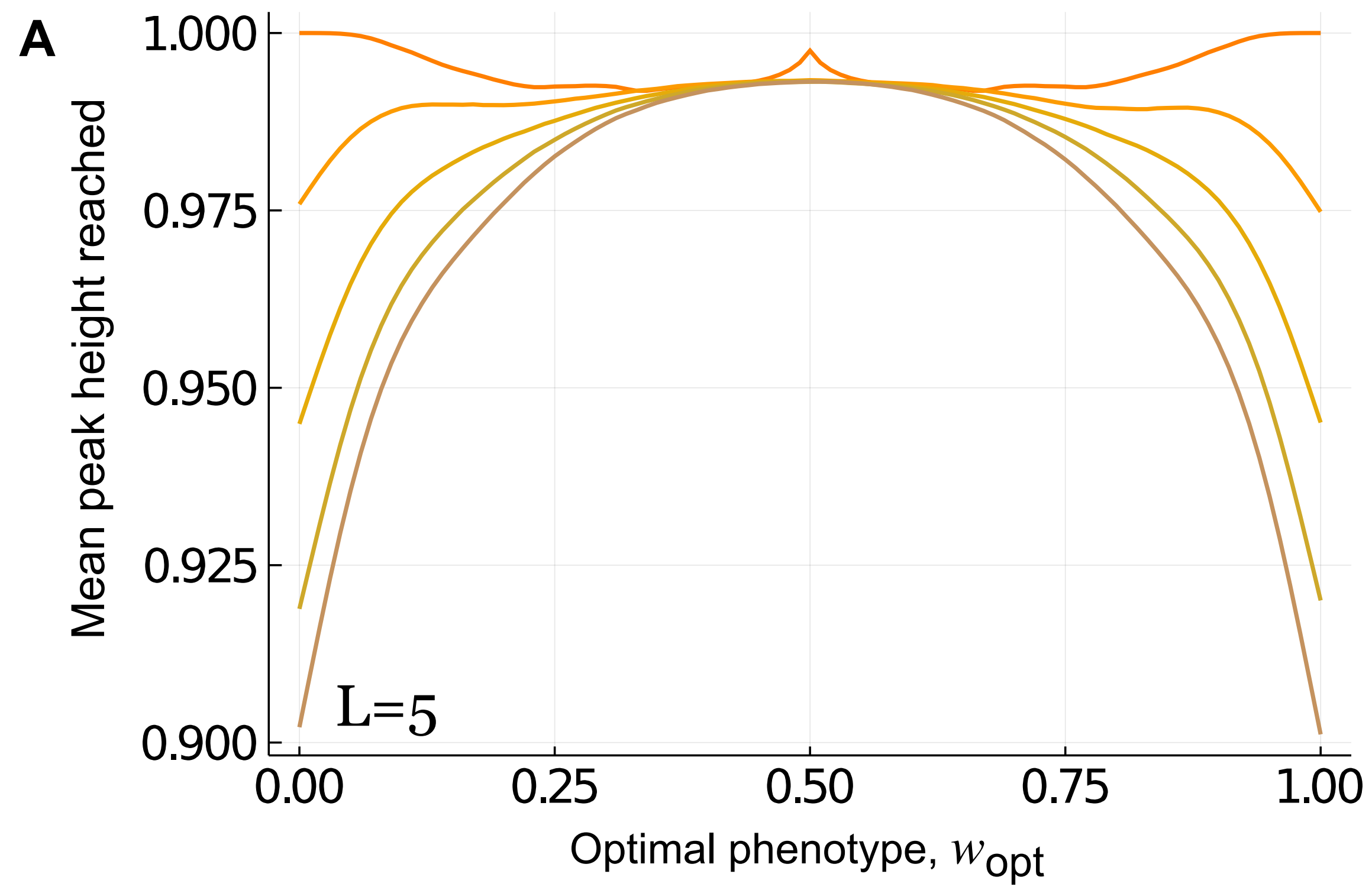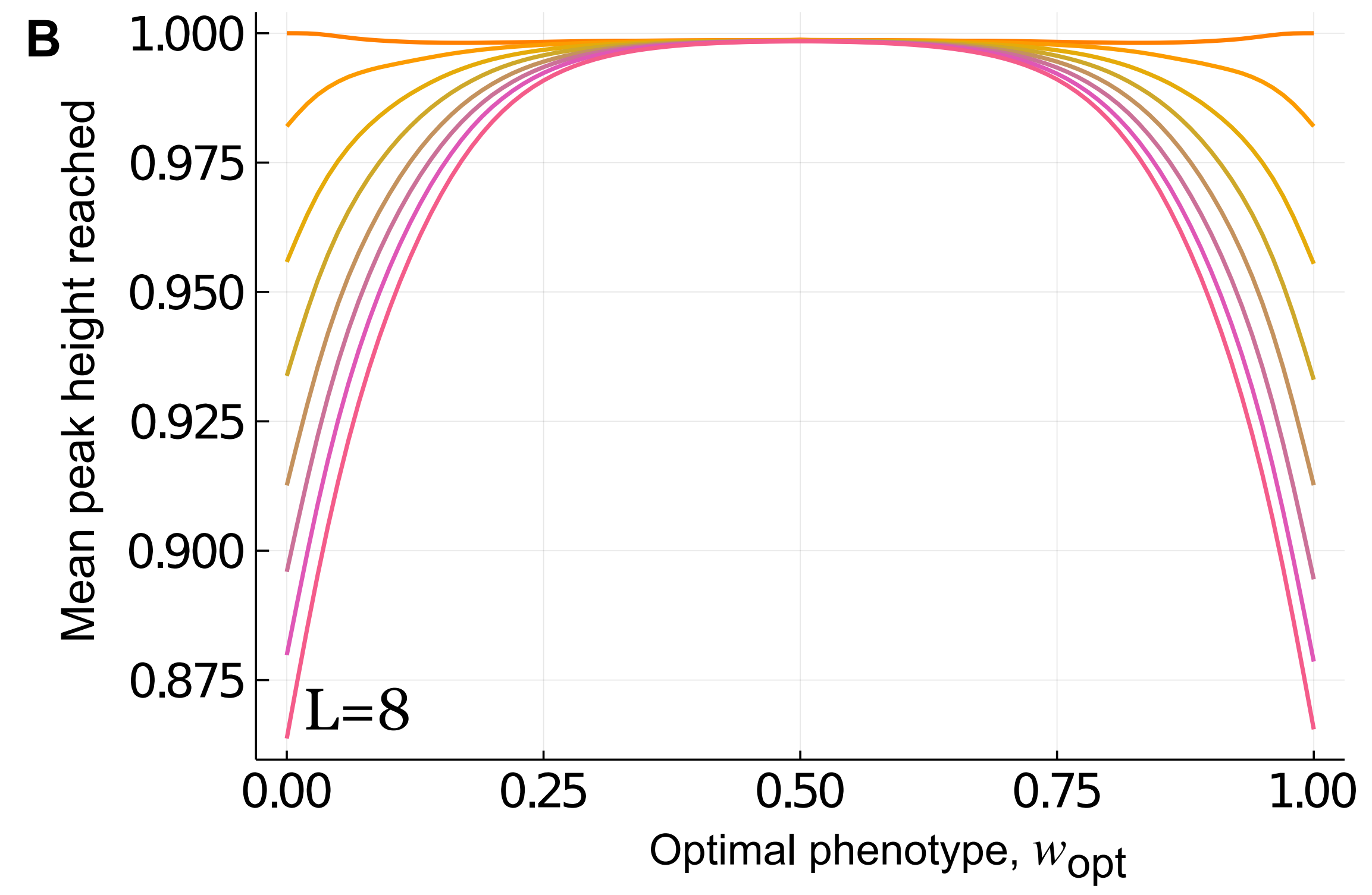

Supplement: S7 Fig — The average height of peaks reached by greedy adaptive walks in NK landscapes with σ = 0.5, shown in relation to wopt, for (A) L = 5 and (B) L = 8, with K = 0…L − 1. (PDF) [file pcbi.1010524.s008.pdf]

$\sigma = 1, \mu = 0.01$

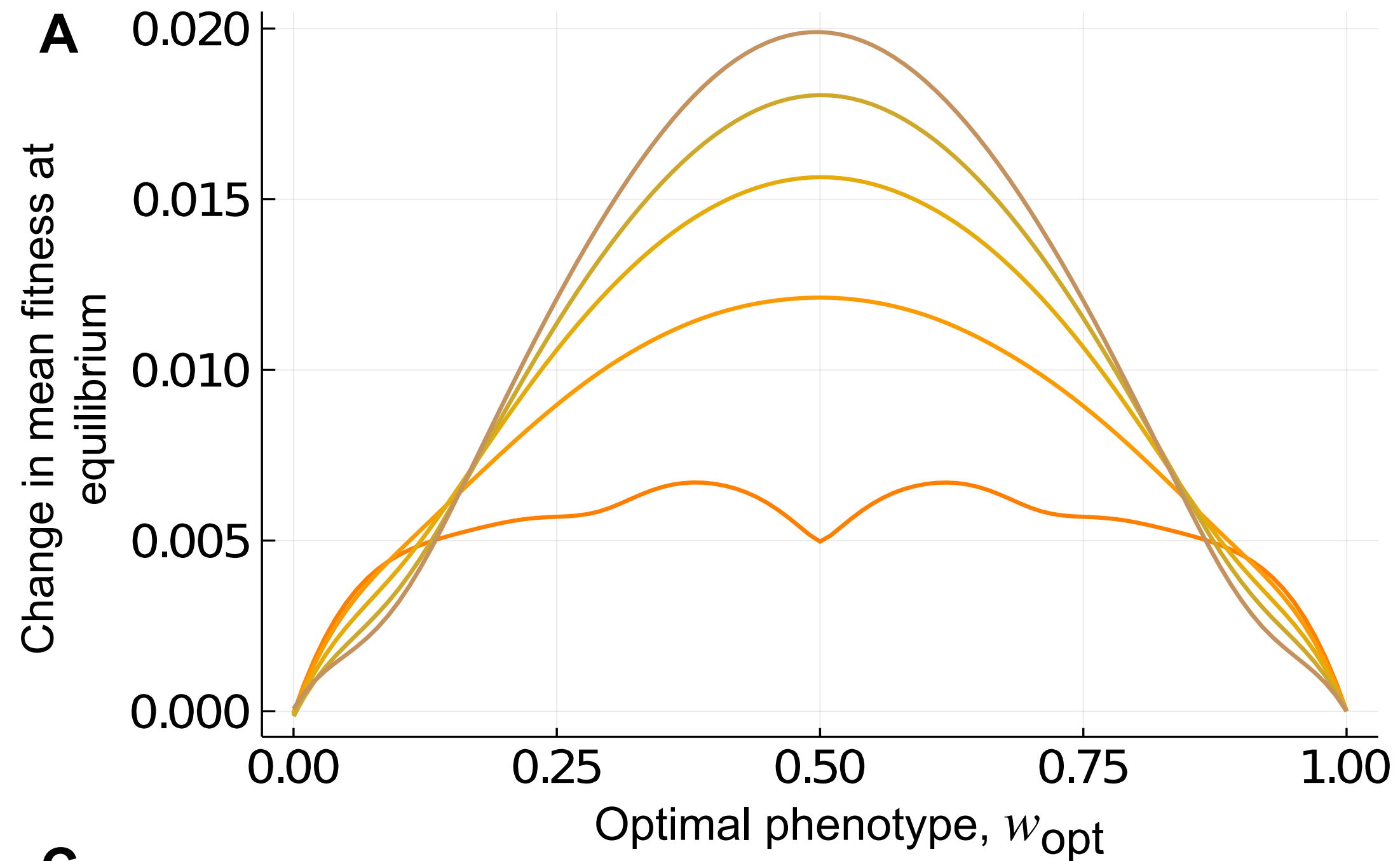

← L=5 →

$\sigma = 0.15, \mu = 0.1$

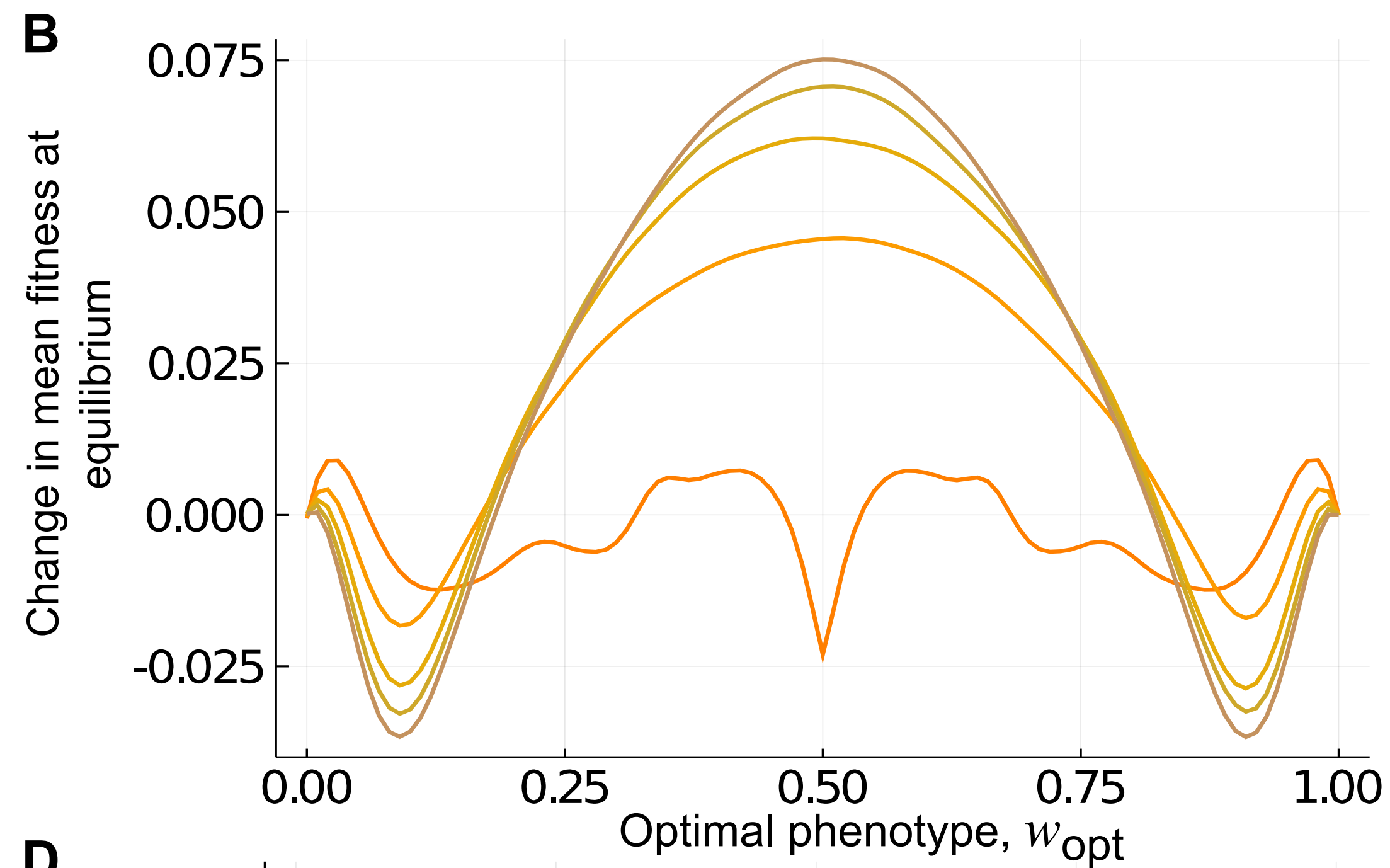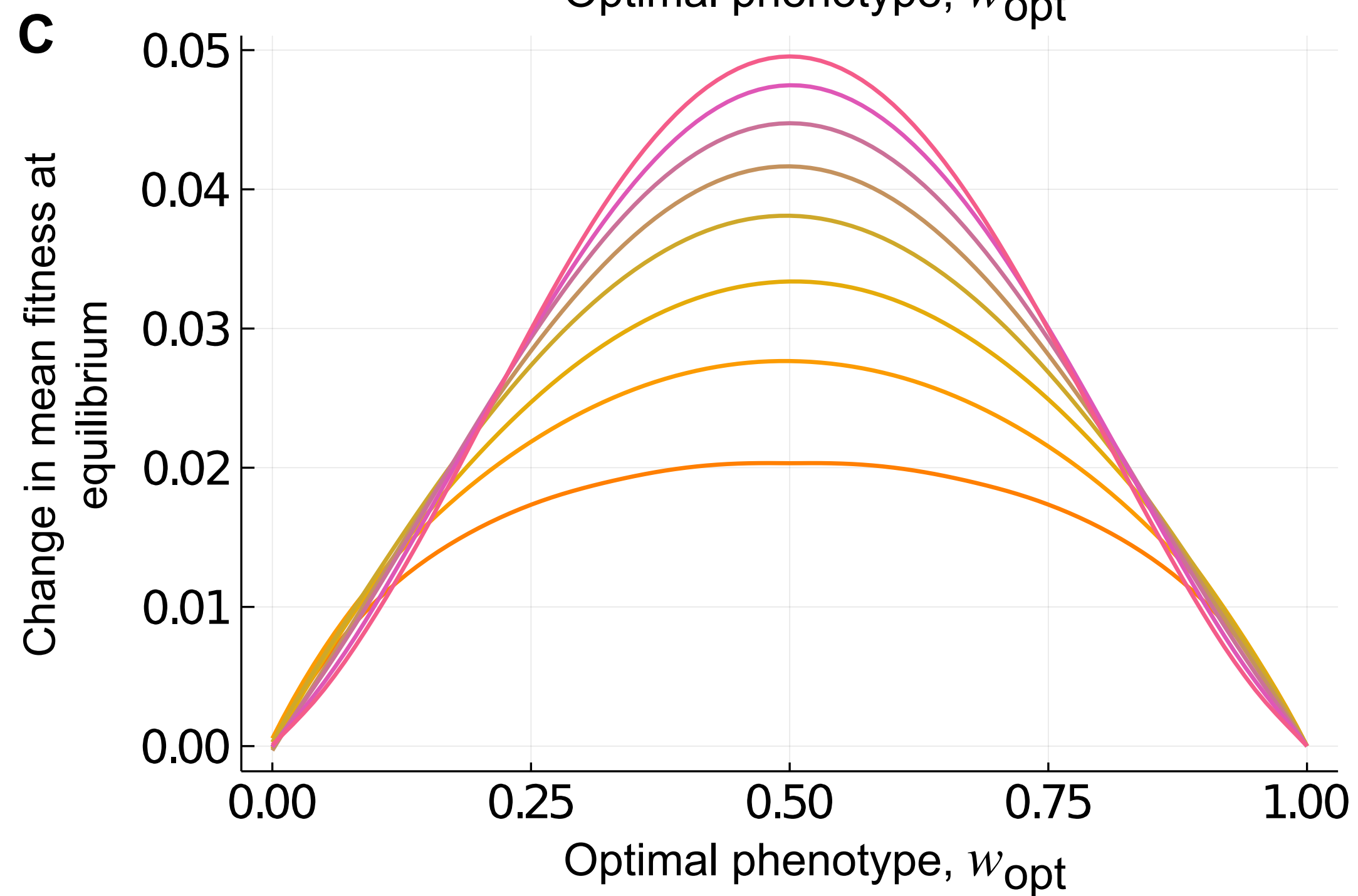

← L=8 →

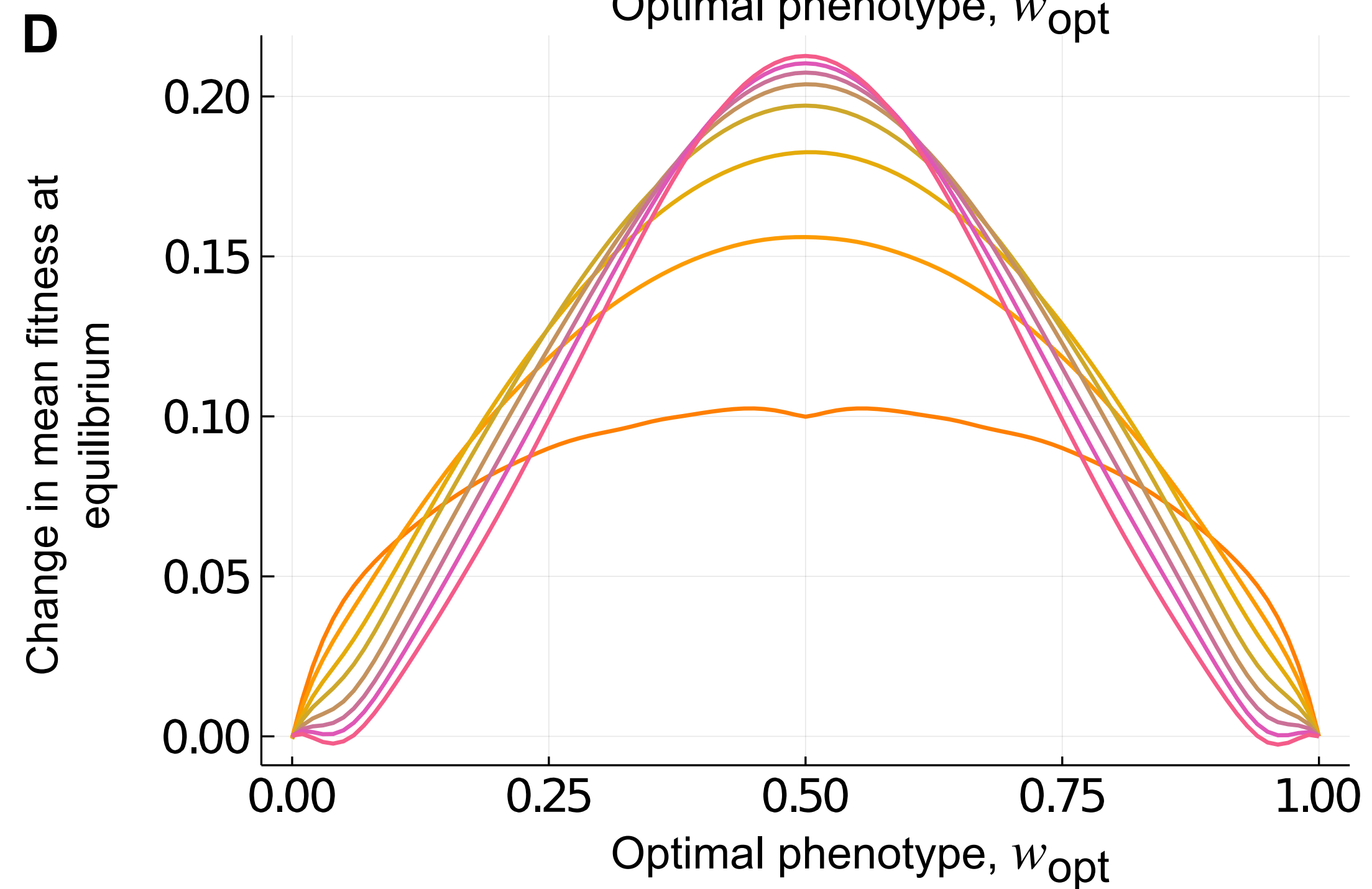

Supplement: S8 Fig — Change in mean fitness at equilibrium for NK landscapes with (A,B) L = 5 and (C,D) L = 8, shown in relation to wopt, for different values of σ and μ. (PDF) [file pcbi.1010524.s009.pdf]

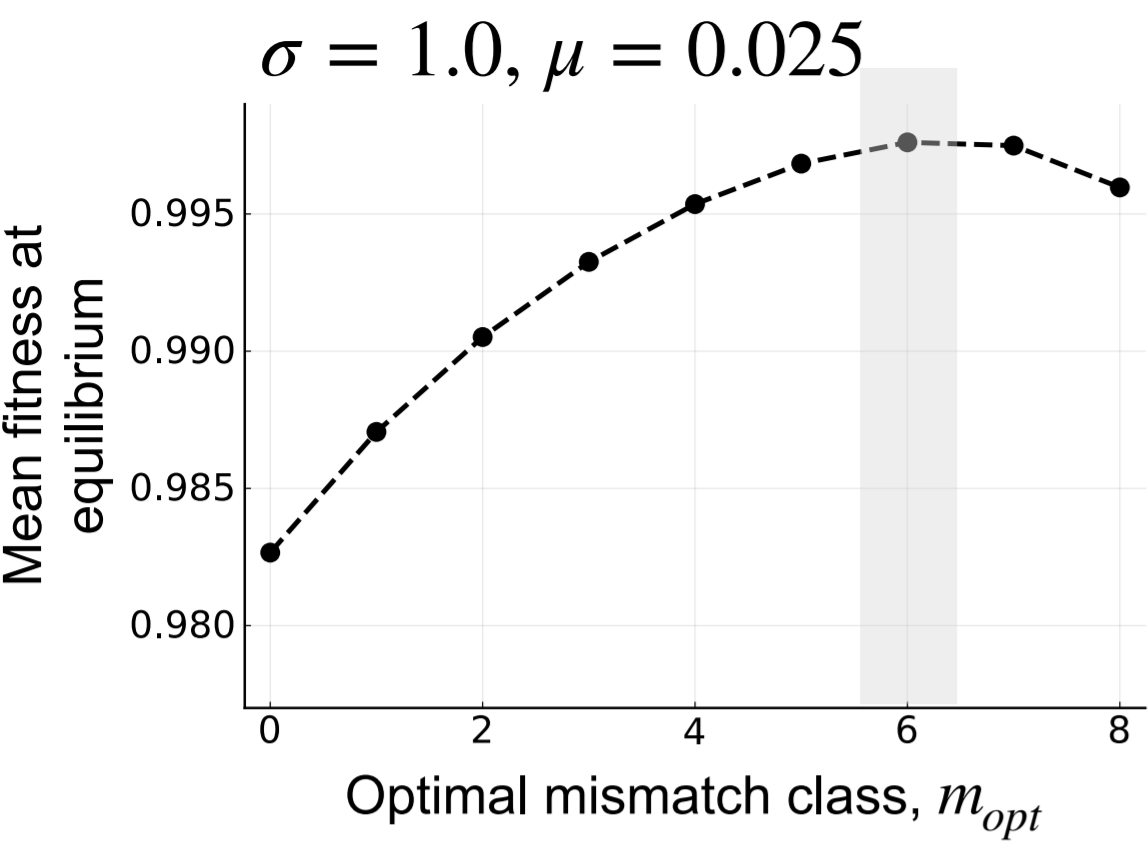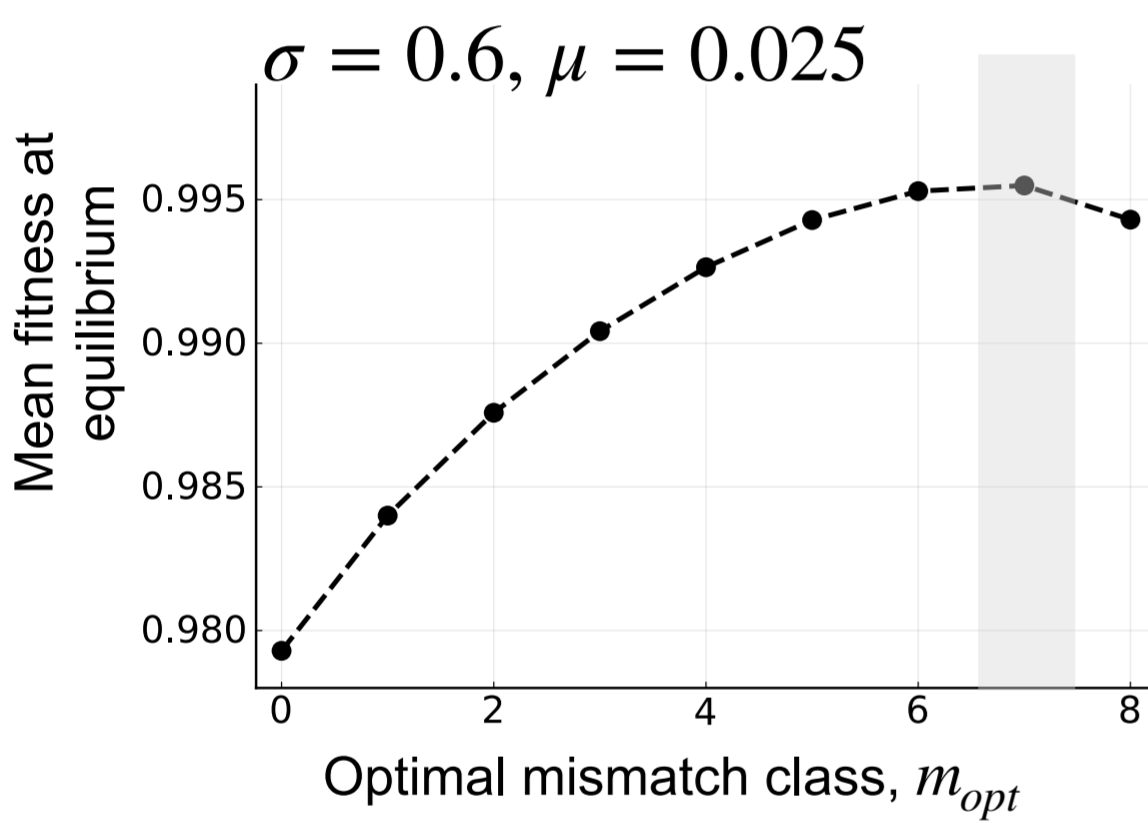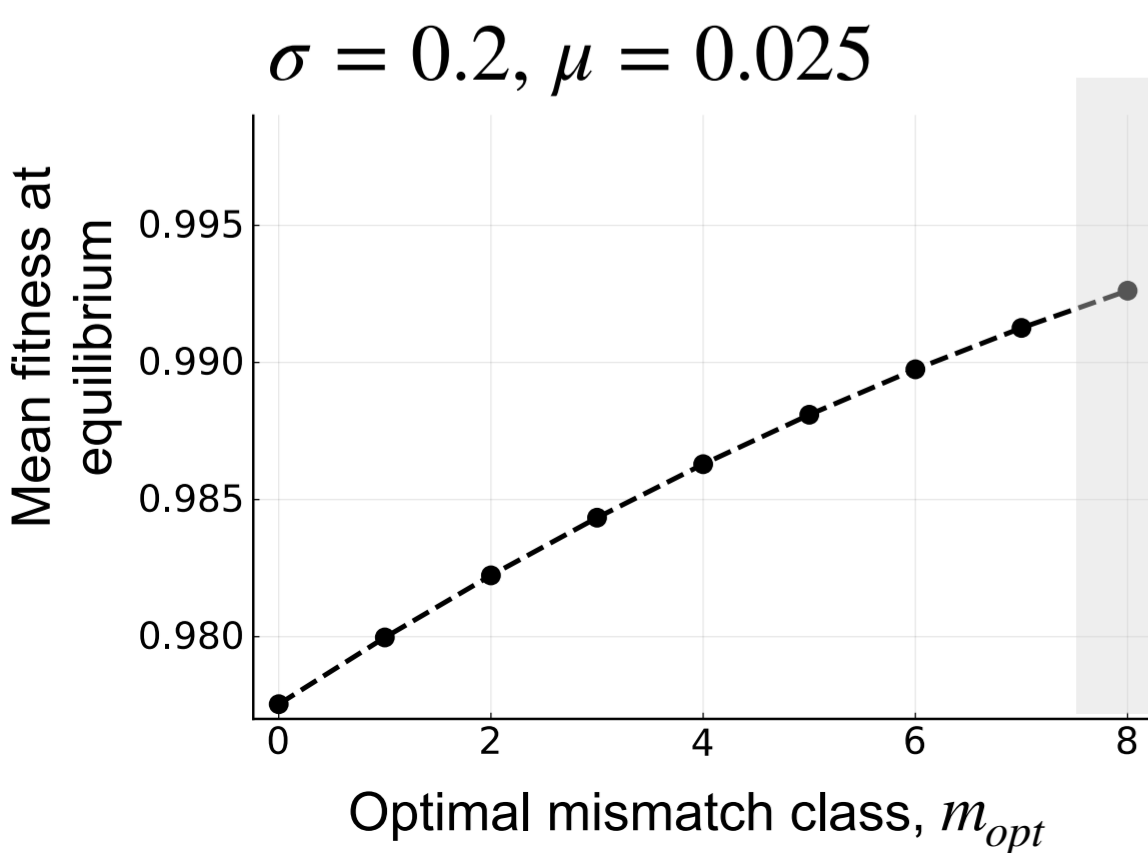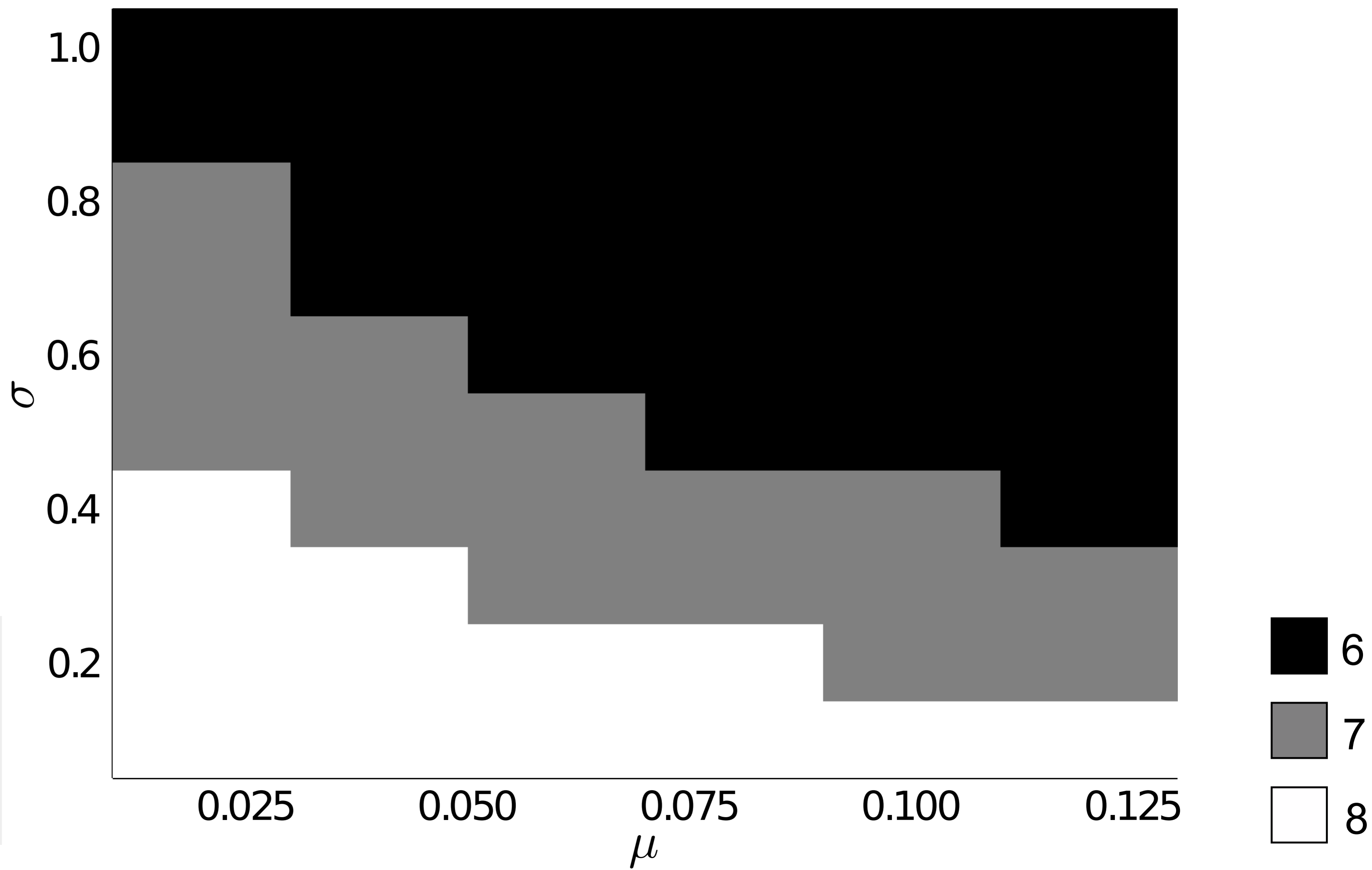

Supplement: S10 Fig — The mismatch class mopt that maximizes mean fitness at equilibrium is shown in relation to the strength of selection σ and the mutation rate μ. The three smaller panels show mean fitness at equilibrium in relation to mopt for three combinations of σ and μ. The value of mopt that maximizes mean fitness is indicated with a gray rectangle. (PDF) [file pcbi.1010524.s011.pdf]

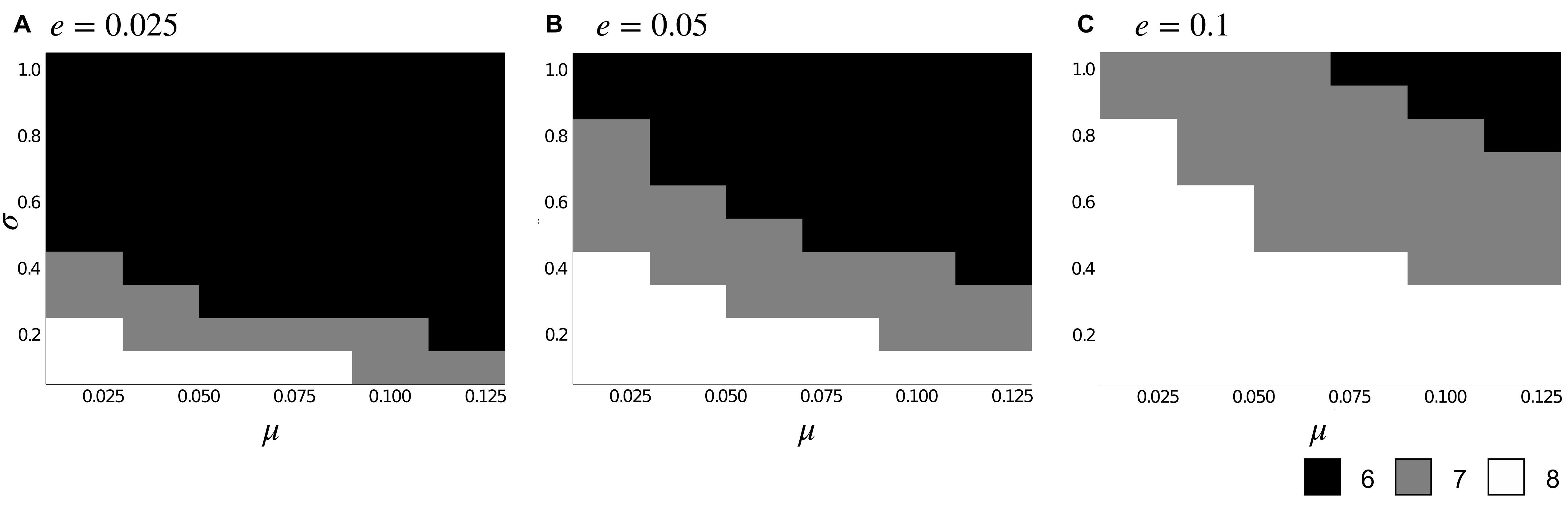

Supplement: S11 Fig — The mismatch class mopt that maximizes mean fitness at equilibrium is shown in relation to the strength of selection σ and the mutation rate μ for three different values of the mismatch penalty e: (A) e = 0.025, (B) e = 0.05 and (C) e = 0.1. (PDF) [file pcbi.1010524.s012.pdf]

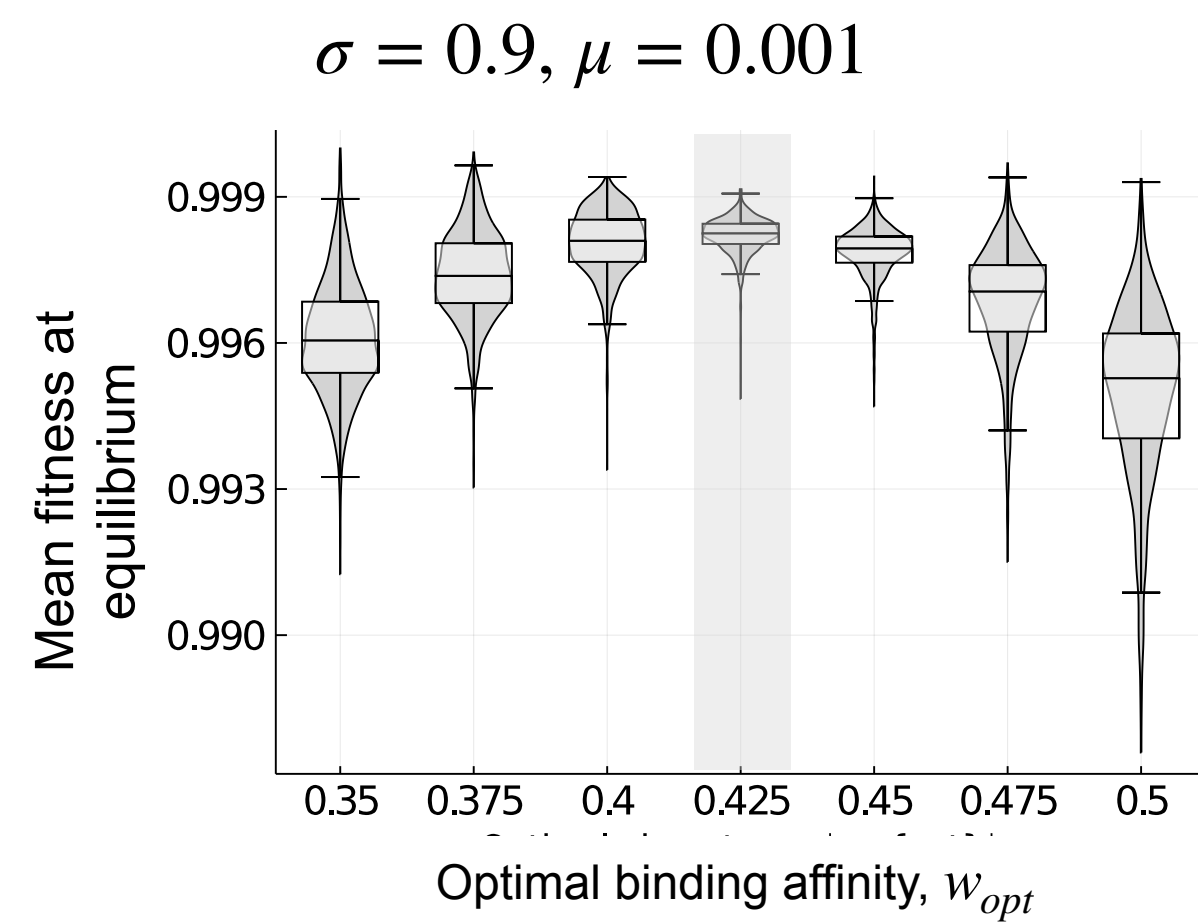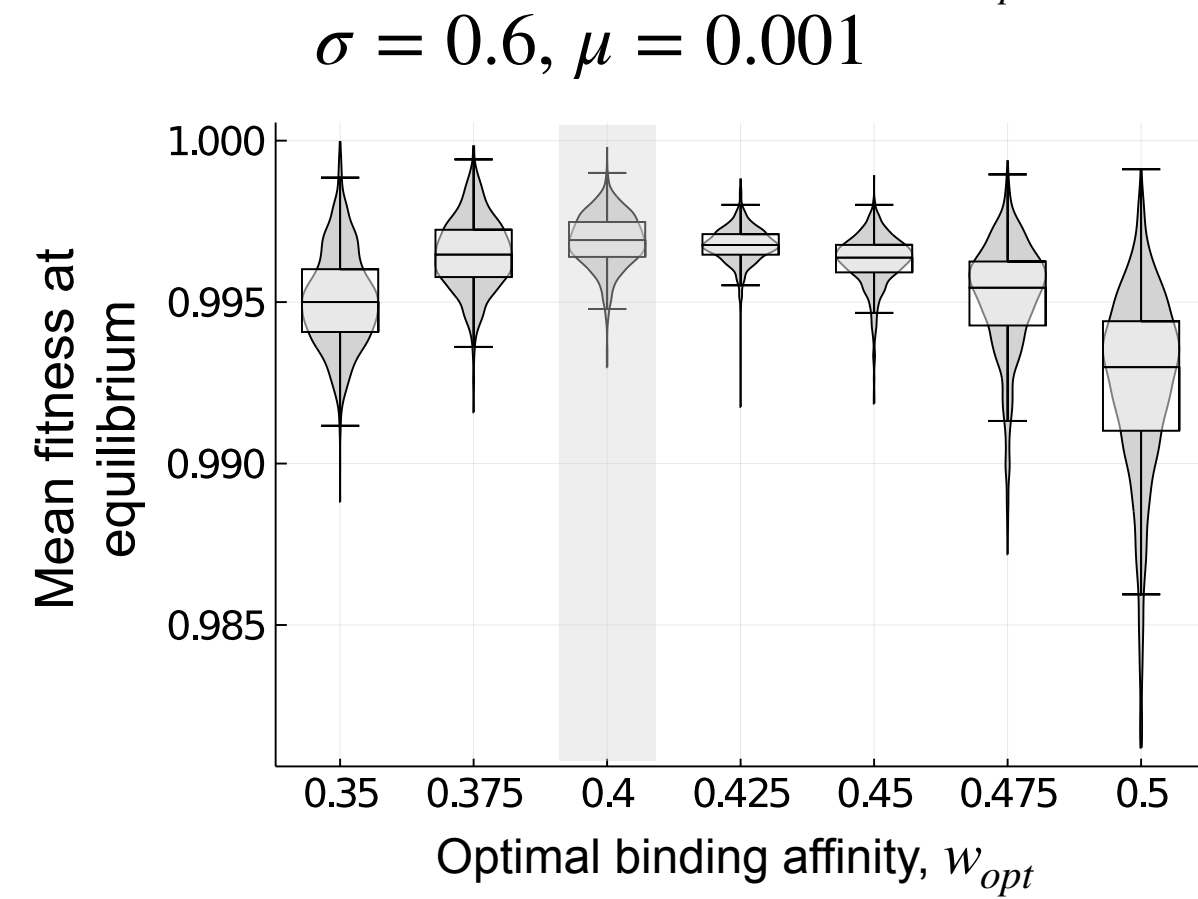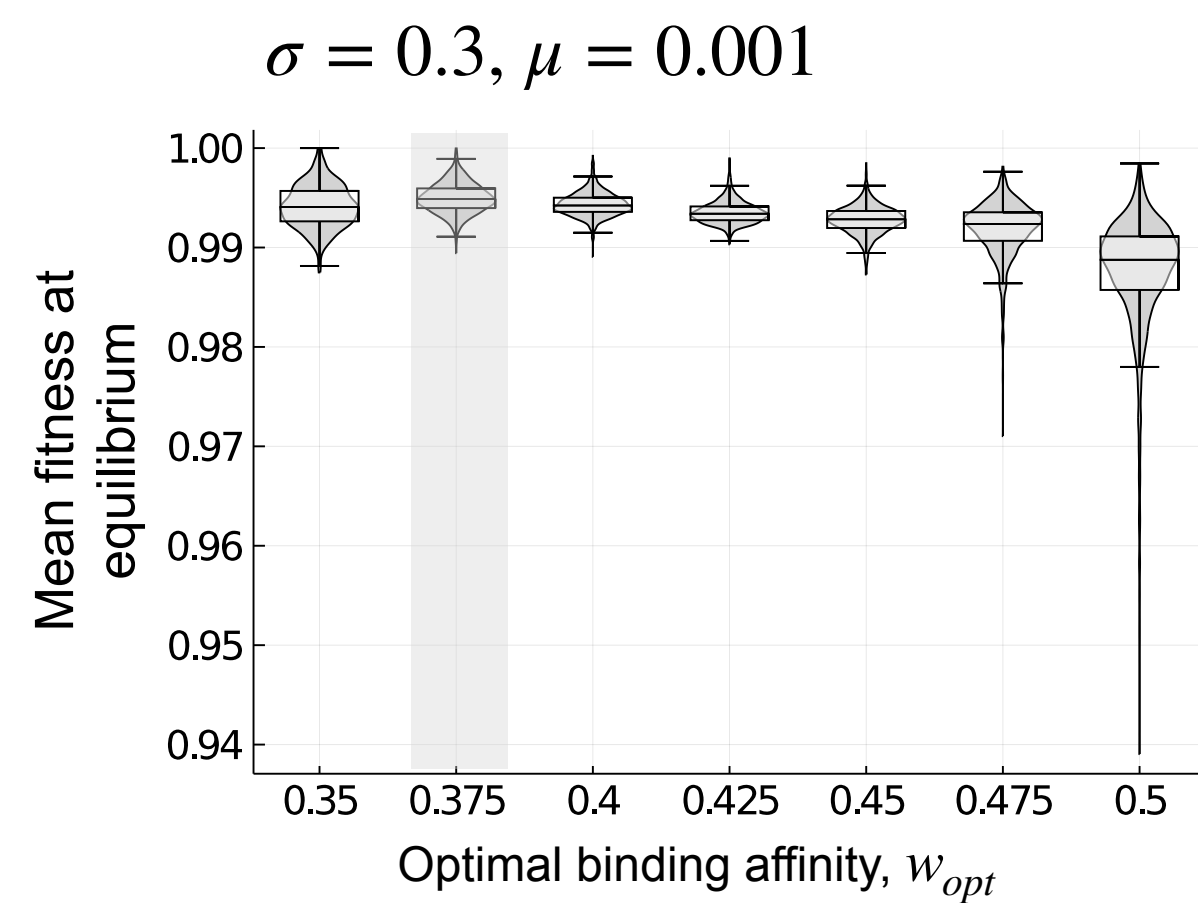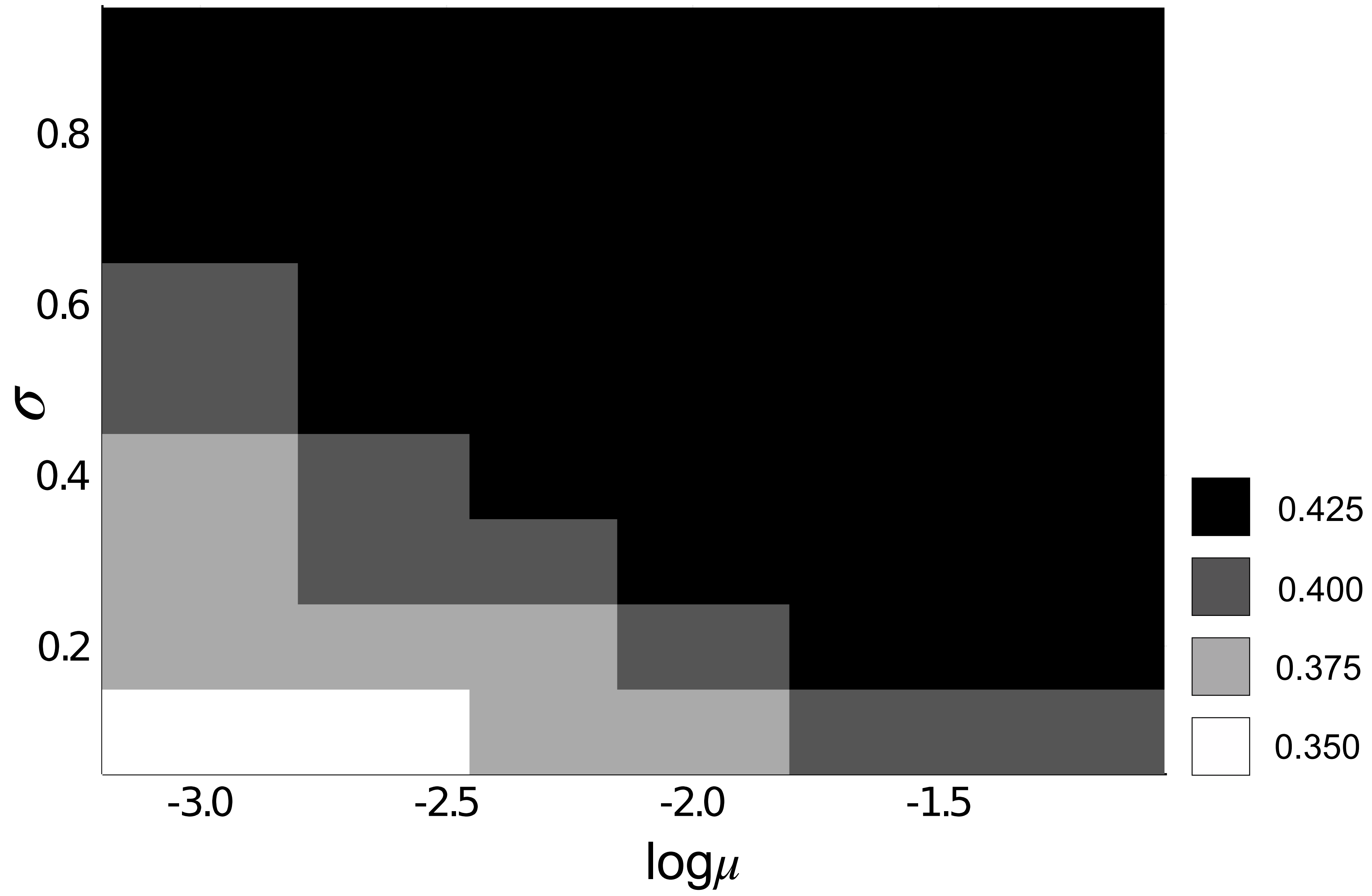

Supplement: S12 Fig — The binding affinity wopt that maximizes mean fitness at equilibrium is shown in relation to the strength of selection σ and the logarithm of the mutation rate (log μ) for the 1,137 empirical landscapes. The three smaller panels show the distributions of mean fitness at equilibrium as violin plots, in relation to wopt, for three combinations of σ and μ. Box-and-whisker plots show the 25–75% quartiles. The value of wopt that maximizes mean fitness is indicated. These results are robust to perturbations of the fitness values (Methods), with the exception of two parameter combinations—σ = 0.1, μ = 0.001 and σ = 0.1, μ = 0.0025. For these parameter combinations, the binding affinity wopt that maximizes mean fitness at equilibrium changes from 0.35 to 0.385 (on average) and 0.375 respectively. (PDF) [file pcbi.1010524.s013.pdf]
