# Supplementary material for: On the incongruence of genotype-phenotype and fitness landscapes
Source: PLoS Comput Biol. 2022 Sep 19;18(9):e1010524. doi: 10.1371/journal.pcbi.1010524 (PMC9521842; doi:10.1371/journal.pcbi.1010524)
Supplement: S9 Fig — (A) Local peaks on which the adaptive walks terminate tend to be nearly as tall as the global peaks in the 1,137 empirical landscapes. Violin plots show the distribution of the fractional height of local peaks reached by greedy adaptive walks, relative to the height of the global peak, for each optimal binding affinity wopt. (B) Violin plots show the distribution of the fraction of walks terminating on the global peak, for each optimal binding affinity wopt. (PDF) [file pcbi.1010524.s010.pdf]

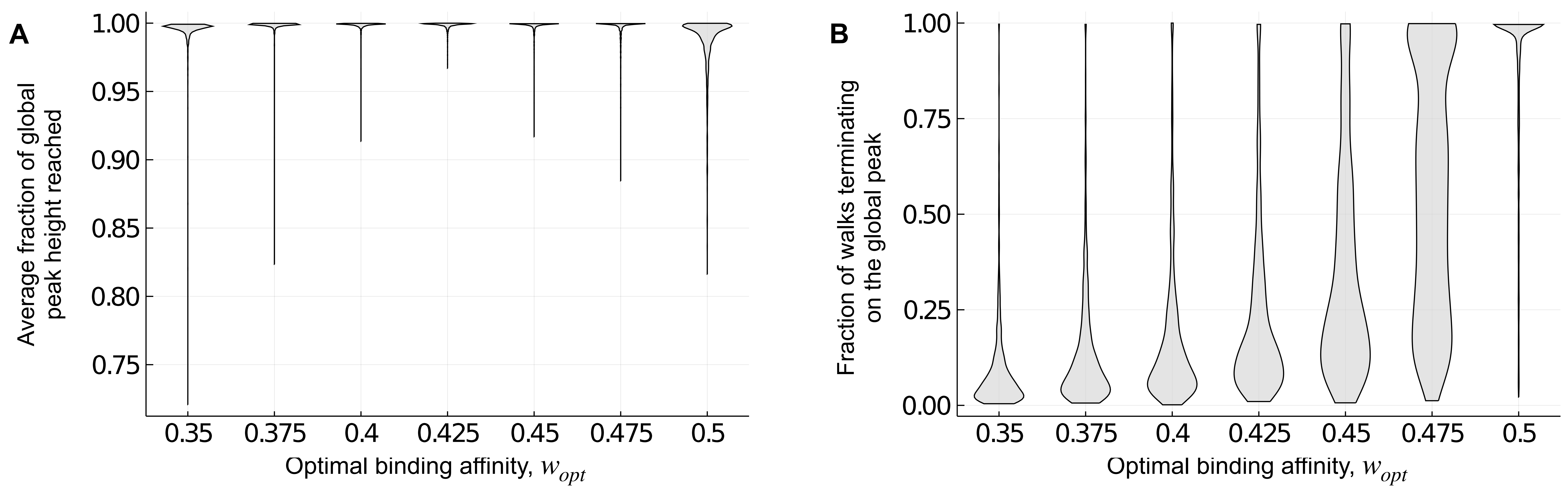

A
Average fraction of global peak height reached
1.00
0.95
0.90
0.85
0.80
0.75
0.35
0.375
0.4
0.425
0.45
0.475
0.5
Optimal binding affinity, w_{opt}
B
Fraction of walks terminating on the global peak
1.00
0.75
0.50
0.25
0.00
0.35
0.375
0.4
0.425
0.45
0.475
0.5
Optimal binding affinity, w_{opt}
