# Supplementary material for: On the incongruence of genotype-phenotype and fitness landscapes
Source: PLoS Comput Biol. 2022 Sep 19;18(9):e1010524. doi: 10.1371/journal.pcbi.1010524 (PMC9521842; doi:10.1371/journal.pcbi.1010524)
Supplement: Attachment — Submitted filename: ResponseLetter_Final.pdf [file pcbi.1010524.s014.pdf]

Dear Dr. Bollenbach & Dr. Mustonen,

Thank you for inviting the resubmission of our manuscript “On the incongruence of genotype-phenotype and fitness landscapes” (Tracking number PCOMPBIOL-D-22-00693) to *PLoS Computational Biology*.

We would first like to thank you and the two reviewers for the constructive comments, which helped us improve our manuscript significantly. For example, we have now demonstrated that our results concerning peak accessibility are not simply a consequence of the existence of plateaus in the genotype-phenotype landscapes (reviewer 1) and we have added the important corollary to our work that under arbitrary unimodal phenotype-fitness maps, a fitness landscape will always be congruent with its underlying genotype-phenotype landscape when that landscape contains only simple sign epistasis motifs (reviewer 2).

Below, we respond to each comment point by point. The comments made by the reviewers are repeated verbatim in blue font, our responses are in black font, and changes to the manuscript are in quoted black italic font. We also provide a version of our manuscript in which all changes to the text are tracked.

We thank you again for considering our work for publication in *PLoS Computational Biology*.

Best Regards,
Joshua L. Payne

Comments to the Authors:
Please note here if the review is uploaded as an attachment.
Reviewer #1: The manuscript “On the incongruence of genotype-phenotype and fitness landscapes” investigates how the ruggedness of fitness landscape is related to the underlying genotype-phenotype landscape when different selective pressures are applied. By using multisite biallelic genotype-phenotype landscapes and Gaussian phenotype-to-fitness map centered around phenotypic optimum, authors derive analytical relations for local and global properties of resulting fitness landscapes. The results are verified computationally for genotype-phenotype landscapes in the NK model and in the experimentally obtained dataset containing binding affinity of TF to DNA. The important finding is that selection for low and intermediate phenotypic values significantly increases ruggedness of the landscape, but interestingly the evolutionary accessibility of global peak is not affected.

The question of how different selective pressures modify both level of epistasis and number of adaptive peaks when genotype-phenotype landscape is mapped to the fitness landscape is a very interesting one with potential implications in both theoretical and experimental studies of biological evolution. The manuscript provides a very valuable contribution in terms of identifying different factors affecting local and global measures of ruggedness when mapping genotype-phenotype landscape into fitness landscape. I highly appreciate supporting computational results with analytical calculations that could guide our intuition about system behavior. The paper is well written, technically sound and results are well presented. My only major concern, is how the multi-allelic landscape is mapped to bi-allelic landscape, and whether this mapping is not biasing the evolutionary accessibility of resulting fitness landscape. Still, I expect this concern could be addressed.

In summary, I find that this work deserves a broad dissemination and will be of interest for the PLoS Computational Biology audience.

We thank the reviewer for their careful reading and overall positive assessment of our manuscript.

Major issue

1. The multi-allelic genotype-phenotype landscape that mimics energy landscape of TF-DNA interactions is transformed into bi-allelic landscape through the threshold model. The threshold model results in genotype-phenotype landscape with large plateaus corresponding to low and intermediate phenotypic values. If now one phenotypic value corresponding to one of these plateaus is selected as most optimal it is not surprising that although the landscape is very rugged the optimal peaks can be reached with very short adaptive walks.

However, in more realistic approach the energy landscape of TF-DNA would likely not have these plateaus, but rather a spread of phenotypic values (see Mustonen et al. 2008, cited as [43]), with empirical distribution of these energies resembling rather a continuous bulk of non-specific bindings and additional peak for specific bindings. My objection with the current mismatch model is that applying a fitness map to the idealized plateau-like landscape, could be qualitatively different to applying the same fitness map to a landscape in which plateaus are not present. In practice one could introduce noise to phenotypic values in plateau-like landscape, or consider more fine-grained distinction of binding energies to introduce larger number of discrete phenotypic levels. The question would be whether fitness map will still result in fitness landscape with short adaptive walks toward the global fitness optimum?

I would appreciate at least a discussion of such case, as my impression is that current conclusion about no change in accessibility of adaptive peaks is simply a consequence of the plateau-like mapping introduced by threshold model.

The reviewer seeks additional guidance regarding the influence of plateaus on the length of adaptive walks. We have followed the reviewer's suggestion to "consider a more fine-grained distinction of binding energies to introduce a larger number of discrete phenotypic values." We did this by re-running our analyses on the 1,137 empirical landscapes, but this time setting the noise threshold δ to zero. This makes all phenotypic values differentiable, and thus ensures the spread of phenotypic values mentioned by the reviewer, resulting in landscapes without plateaus.

We observe the following:

- Expectedly, the change in number of peaks in these landscapes increases for every w_{opt}, relative to the landscapes generated when δ>0. However, the general trend remains the same (compare to Fig. 6D):

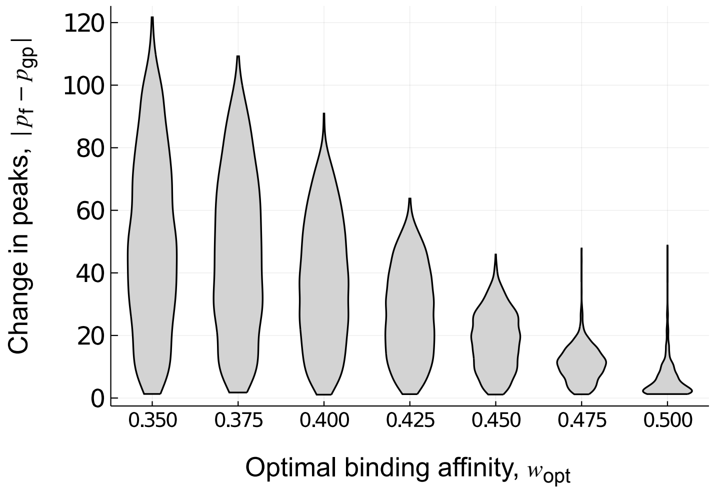

- The average length of an adaptive walk is now minimised while selecting for the lowest binding affinity and monotonically increases as w_{opt} increases (compare to Fig. 7B). This too is expected, because in the absence of plateaus, we expect the length of the adaptive walks to be inversely correlated with the number of peaks:

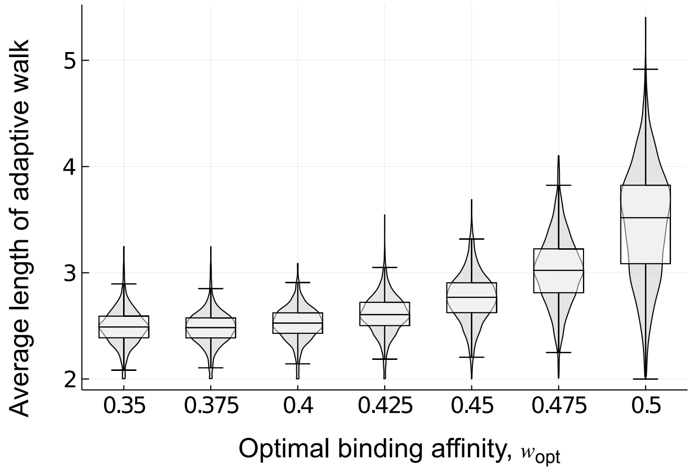

- However, the fraction of walks terminating on the global peak, and the mean peak height reached, show the same trends as in our original analysis (compare to Supp Fig. 9):

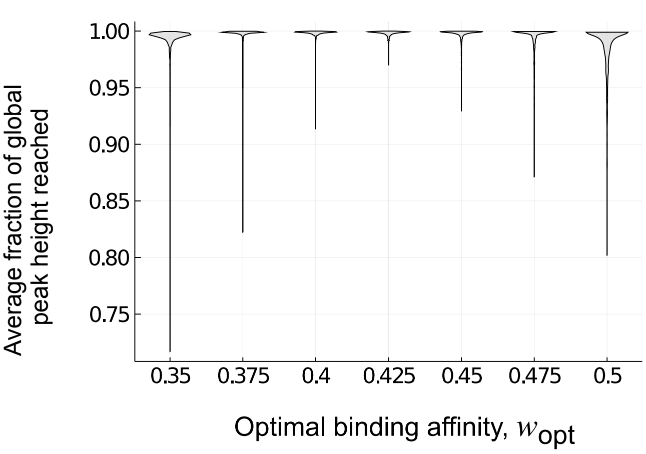

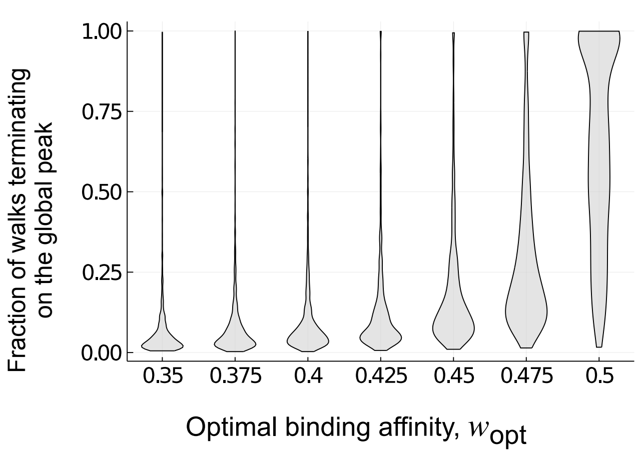

Thus, our results concerning peak accessibility are not simply a consequence of the existence of plateaus — selecting for intermediate phenotypic values creates fitness landscapes that have higher average fitness and thus we expect the walks to terminate at higher peaks. We had previously provided this explanation in Supplementary Material, Note 2 for the case of NK landscapes, but this was clearly too late. We have now added the following forward reference to the main text (lines 459-465):

"*We note that in the absence of plateaus (i.e., when the noise threshold is zero),* $\langle l \rangle$ *increases monotonically with* w_{opt}*, because in this case,* $\langle l \rangle$ *is inversely correlated with the number of peaks in the fitness landscape, which decreases monotonically with* w_{opt}*. Regardless of the noise threshold, when selecting for low or intermediate phenotypic values, most walks terminate at a local, rather than the global fitness peak. However, these local peaks tend to be nearly as high as the global peak, especially when selecting for intermediate phenotypic values (Supplementary Fig. 9). These results hold for all noise thresholds, and are therefore not a consequence of the existence of plateaus in the genotype-phenotype landscapes (see Supplementary Material, Note 2 for explanation).*"

Further, we highlight that plateaus are an important and commonly observed topographical feature of adaptive landscapes, not only in transcription factor-DNA interactions [Aguilar-Rodríguez et al. *Nat Eco Evo*, 2017], but also in other systems [Ahnert SE., *J. R. Soc. Interface,* 2017]. The conclusions we draw here should therefore be broadly applicable.

Minor issues
2. lines 167-168, please give explicitly the cut-off for zero fitness, instead of "within computer precision".

We have followed the reviewer's request and specified the explicit cut-off (line 169):

"within computer precision i.e. any fitness $< 5 \times 10^{-324} \approx 0$*"*

3. Eq 5 and lines 436-439, the properties of accessible pathways in multi-allelic landscapes were studied in Zagorski et al., PLoS Comp Bio 2016, indicating that increasing number of alleles would increase the average length of mutational pathways towards global optimum. Does it hold for eq. 5?

We thank the reviewer for pointing out this important paper, which we now cite. It concerns the influence of indirect paths on global peak accessibility, whereas our Eq. 5 quantifies the mean length of *direct* paths to the global peak, which is always accessible due to the topographical properties of landscapes generated using the mismatch model. Thus, our Eq. 5 cannot be directly compared to the results of Zagorski et al.. Since under the mismatch model the peaks are broad (and contain $\binom{L}{k} \cdot (a-1)^k$ sequences in total), increasing the number of alleles *a* will increase the number of sequences in peaks for any mismatch class *k*, and thus the average length of an adaptive walk will decrease with *a*.

In response to this comment, we have added the following text and citations (Zagorski et.al, *PLoS Comp, Biol.*, 2016; Wu et al., *eLife*, 2016) (lines 402-405):

"Whereas previous work has shown that peak accessibility increases with alphabet cardinality (a) *due to the existence of indirect paths*[10,80]*, we show below that despite this increased accessibility, results qualitatively similar to the bi-allelic case* (a*=2) also hold for the multi-allelic case of the mismatch model and of the empirical landscapes of TF-DNA interactions* (a*=4)."*

4. figure 7 caption, there are no "Closed symbols and dashed line" in figures 7B and 7D. Please remove or correct figures.

We have corrected the caption accordingly.

Reviewer #2: The topic of the paper are the changes in landscape properties that occur when a genotype-phenotype landscape is combined with a nonlinear phenotype-fitness map. Specifically, the authors consider a one-dimensional real-valued phenotype that is mapped to fitness via a nonlinear function with a unique optimal phenotype value. The primary motivation for the work is the perception that "genotype-phenotype landscapes are often used as a proxy for fitness landscapes", and that this may be potentially misleading. While I agree that this problem exists to some extent (mainly because organismal fitness is oftentimes a complex, multidimensional construct), I feel that it is also something of a straw man, since most workers in the field are very well aware of the distinction between the two concepts.

We agree with the reviewer that most workers in the field are well aware of the distinction between a genotype-phenotype landscape and a fitness landscape. For example, in their

seminal paper “A comprehensive, high-resolution map of a gene’s fitness landscape” (*Mol. Biol. Evol.*, 2014), Firnberg et al. write, “we are referring to “gene fitness” as a phenotypic signature - the suitability of a gene to provide a particular function. Thus, a gene fitness landscape might also be referred to as a phenotypic landscape.” Nonetheless, our statement that “genotype-phenotype landscapes are often used as a proxy for fitness landscapes to study the dynamics and predictability of evolution” remains valid. For example, the phenotype of antibiotic resistance is commonly used as a proxy for fitness to study the dynamics and predictability of evolution [Schenk et al., *Mol. Biol. Evol.*, 2013; Palmer et al., *Nat. Comm.*, 2015].While this phenotype may be a useful proxy in controlled laboratory conditions, its relation to fitness in the wild is less clear, because antibiotic concentrations vary substantially and resistance is costly.However, the reviewer’s point is well taken - we do not believe or wish to suggest that workers in the field are unaware of the distinction between a genotype-phenotype landscape and a fitness landscape. We have therefore added the following (lines 62-64):

“While we do not doubt that workers in the field are well aware that the topographical properties of a fitness landscape can differ from those of its underlying genotype-phenotype landscape, a systematic study of these differences is lacking.”

For the same reason, I'm not really happy with the title of the paper (though I know that it has already been modified compared to the bioRxiv version of the manuscript). The authors address an important problem, viz., the transformation properties of multidimensional phenotypic epistasis under nonlinear phenotype-fitness maps, and I think this could be better reflected in the title.

We sympathise with this comment, because we too liked our old title. However, we received the useful feedback on our *bioRxiv* preprint that our use of the term “transformability” may be confusing to some readers. The reason is that in mathematics, the term “transformable” refers to objects that *do not* change upon the application of a topological transformation. In contrast, our interest is in the topographical *differences* between a genotype-phenotype landscape and a fitness landscape, upon application of a phenotype-fitness map. To avoid this potential confusion, we chose to remove any use of the term “transformability” or “transformable.” In choosing the manuscript’s current title, we sought generality, because the analyses we perform can be applied to any pair of genotype-phenotype and fitness landscapes, not only those related by the Gaussian phenotype-fitness map we chose to study (see also our response to the reviewer’s first specific point below). We would therefore like to keep our current title, and we kindly ask for the reviewer’s understanding.

For the specific case where phenotypic epistasis is absent (and therefore the nonlinear phenotype-fitness map is the sole source of epistasis for fitness) this problem has been addressed extensively (in the context of Fisher's geometric model and elsewhere), and the authors cite a number of studies in this direction, but the generalization to epistatic genotype-phenotype landscapes is novel and timely.

I am generally in favor of publication of the manuscript in PLoS Computational Biology, but ask the authors to address the following specific points prior to acceptance.

We thank the reviewer for their overall positive assessment of our manuscript and for the comments they raised, which we address below.

1. Related to the previous remark, I think the authors should emphasize that their analysis is not restricted to nonlinear phenotype-fitness maps, but applies whenever one phenotype is mapped nonlinearly to another. In such a situation, the analysis of the resulting landscape can provide information about the underlying phenotypic architecture. As an example, Zwart et al. [Heredity 2018] concluded from an analysis of a genotype-resistance landscape for synonymous mutations in an antibiotic resistance enzyme that the underlying phenotype cannot be one-dimensional.

We thank the reviewer for raising this point. We had previously highlighted that our analysis is not restricted to nonlinear phenotype-fitness maps, by writing in our discussion that *"because our measures of incongruence can be applied to any pair of landscapes so long as they are defined over the same set of genotypes, they may shed light on the kinds of topographical alterations induced by fluctuating environmental factors, such as DNA methylation or the presence of protein partners on transcription factor-DNA interactions."*

However, we agree with the reviewer that this should be further emphasised, so we have added the following text to the discussion (lines 546-547):

"Moreover, because our measures of incongruence can be applied to any pair of landscapes so long as they are defined over the same set of genotypes, they are also applicable whenever a phenotype is mapped non-linearly to another phenotype."

Regarding the inference of the underlying phenotypic architecture from the topographical properties of a fitness landscape, the phenotype-fitness map we study is not invertible, so we can only make such inferences in limited cases. We feel this is an important point to make, so we have added the following (lines 548-555):

"Ideally, we would be able to make inferences about phenotypic architecture based on the topographical properties of higher-level phenotypic or fitness landscapes -- as was previously done for an antibiotic resistance phenotype[114]. However, because the phenotype-fitness map we study is not invertible, we can only make such inferences in limited cases. For instance, when the fitness landscape is single-peaked, we can make the probabilistic inference that the underlying genotype-phenotype landscape is also likely to be single-peaked, because the phenotype-fitness map is more likely to increase than to decrease the number of peaks. In contrast, when the fitness landscape has multiple peaks, we can only infer that the underlying genotype-phenotype landscape does not solely comprise simple sign epistasis motifs. Beyond that, we cannot infer the topographical properties of the underlying genotype-phenotype landscape. It could be smooth or rugged."

2. One of the most remarkable results of the paper is that simple sign epistasis motifs (SSE) cannot be transformed into reciprocal sign epistasis (RSE) under a unimodal phenotype-fitness map. Unless I am mistaken, this has an even more remarkable (and quite counterintuitive) corollary. A well-known result by Poelwijk et al. (JTB 2011) states that a multipeaked fitness landscape has to contain at least one instance of RSE. Together with the new result, this would then imply that a genotype-phenotype landscape where all 2-faces have SSE remains single-peaked under arbitrary unimodal phenotype-fitness transformations [it is straightforward to see that such genotype-phenotype landscapes can be constructed for any L]. If the authors agree with this conclusion, I think it is worth mentioning.

We appreciate this suggestion and agree with the reviewer's conclusion. We now mention this conclusion in the abstract, results and the discussion section:

"For example, selection for low or intermediate phenotypic values does not change simple sign epistasis into reciprocal sign epistasis, implying that genotype-phenotype landscapes with only simple sign epistasis motifs will always give rise to single-peaked fitness landscapes under such selection." (lines 17-19)

"Because reciprocal sign epistasis is a necessary condition for multiple peaks[5], this implies that genotype-phenotype landscapes with only simple sign epistasis motifs will always give rise to single peaked fitness landscapes, using the phenotype-fitness map considered here." (lines 194-196)

"Further, we show that the simple sign epistasis motif cannot be converted into reciprocal sign epistasis, implying that genotype-phenotype landscapes with only simple sign epistasis motifs will remain single peaked and globally congruent to their corresponding fitness landscapes." (lines 485-487)

3. Conceptually, it is not clear to me why the authors use the absolute change in the number of peaks as a measure of "incongruence", rather than just the different p_f - p_gp. Isn't is important to know whether the number of peaks increases or decreases?

It is important, which is why we additionally consider the sign of the change (e.g., in lines 259-266). The reason why our primary analysis uses the absolute change in the number of peaks is that we want to take a meaningful average over many realisations of genotype-phenotype landscapes. If we did not use the absolute value, the positive and negative differences would cancel each other out. Based on this comment, we realise this should be said from the outset, so we have added the following text (lines 220-222):

"We use the absolute value of the change in number of peaks so that we can average over many realisations of genotype-phenotype landscapes. However, since the sign of change is also important, we discuss that as well in the following sections."

4. Figure 4A and lines 225-227: I cannot see any maxima in the figure for L \geq 4, and in fact I think the argument in the SM (Note 1) is flawed. When the optimal phenotype is between two integer multiples of 1/L, there will be peaks in both layers of the cube and there is no reason why the total number of peaks should decline.

We thank the reviewer for expressing this concern, because it shows that our arguments were not sufficiently clear. The key insight that we should have offered is that when the optimal phenotype is between two integer multiples of 1/L, it is impossible for peaks to be in both layers of the cube, because the layers are mutationally connected. Instead, we may have broader but still fewer peaks, because mutationally-connected sequences will be part of the same peak.

However, we feel that this is a minor point that is only leading to confusion and not adding much to our manuscript. Therefore, we have decided to omit this point from the main text, and provide a forward reference to the Supplementary Material, Note 1 (lines 231-232):

"For a detailed explanation of the shape of the curves in Fig. 4A, see Supplementary Material, Note 1."

In the Supplementary Material, Note 1, we have made clear why there should still be a decline in the number of peaks, because mutationally connected layers of the cube cannot lead to distinct peaks (lines 124-130 in the Supplementary Material):

"Next, there is a slight decline due to competition between the single and double mutants as peaks (because these are mutational neighbours and therefore cannot both be peaks), followed by the emergence of the second maxima at $2/L$ when all the double mutants become peaks, and so on. Naturally, because the number of genotypes with n mutations increases as n reaches the value $L/2$, the height of the maxima increases accordingly. Thereafter, we observe a fall in $\langle |p_f - p_{gp}| \rangle$. Therefore, for a genotype of length L, there should be $L-1$ maxima in the plot of $\langle |p_f - p_{gp}| \rangle$ vs. w_{opt}. However, the maxima are closely spaced and therefore merge into one another and cannot be distinguished from each other."

5. As it stands, Eq.(3) makes no sense, because the right hand side diverges with L. This should be rewritten to make it into a proper limit statement.

We thank the reviewer for pointing this out. It was indeed incorrect as written. We're simply providing an approximation that works for large *L*, not a limit statement. We have made the following modification:

$$\langle |p_{\mathrm{f}} - p_{\mathrm{gp}}| \rangle \approx \sqrt{\frac{2^L \cdot (L-1)}{2\pi(L+1)^2}} \text{ for } L \gg 1$$

6. Figure 7B: Where does the enormous variability of the adaptive walk length come from?

It is because the walks are random on non-peak plateaus. We have added the following line to explain this (Figure 7, caption):

"*The large variability of $\langle l \rangle$ at intermediate and high w_{opt} is a consequence of the random diffusion of the population on non-peak plateaus, which results in longer walks.*"

7. Supplementary figure 3: There are analytic results for the mean length of greedy adaptive walks in the HoC and NK-models (e.g., Orr, JTB 2003; Nowak and Krug, J. Stat. Mech. 2015). In particular, the greedy walk length for the HoC model is e-1 \approx 1.7 for large L, which is smaller than the results shown in Fig. S3A and C. Do the authors possibly use a nonstandard definition of walk length where a 'zeroth' step is included?

We thank the reviewer for pointing this out. We were indeed using a nonstandard definition where the 0th step was included. We have modified our analysis to use the standard definition, the results of which are now visualised in an updated Supplementary Figure 3. We have also added the suggested references (line 87).
